# Robust multi-class DDoS and fraud detection using a Context-Adaptive Deep Evolutionary Game Framework with CNN–BiGRU

**DOI:** 10.3389/frai.2026.1928178

**Published:** 2026-09-30

**Authors:** Ashwini V., Priya V.

**Affiliations:** School of Computer Science Engineering and Information Systems (SCORE), Vellore Institute of Technology, Vellore, India

**Keywords:** adversarial robustness, context-aware security, DDoS mitigation, evolutionary game theory, hybrid deep learning, intrusion detection, neuro-evolutionary resampling

## Abstract

Distributed Denial of Service (DDoS) attacks have become a serious threat in modern computer networks, which can commonly be integrated with other types of attacks to evade detection by conventional devices. Existing deep learning-based intrusion detection methods have high identification capability. However, they are also highly susceptible to adversarial attacks, in which even slight perturbations to features can fool the model into misclassification. To address these issues, this study introduced a hybrid deep learning framework. It integrates Convolutional Neural Networks (CNN) and Bidirectional Gated Recurrent Units (BiGRU) to detect diverse types of attacks in real time. CNN extracts spatial interactions to model traffic characteristics, while BiGRU, a recurrent neural network, models temporal dependencies in data flows. This combination enables the model to capture complex spatiotemporal behavioral patterns in network traffic. To address class imbalance, we propose a new Neuro-Evolutionary Resampling Algorithm (NERA). This method uses neural density estimation and evolutionary optimization to generate balanced, noise-filtered training samples. Additionally, a Context-Adaptive Deep Evolutionary Game Framework (CADEGF) is designed to improve resistance to attacks. It does this by simulating interactions between attackers and defenders. It also adjusts detection thresholds using game-theoretic ideas. Experimental results show that this approach achieves high classification accuracy, effectively detects minority classes, and highlights trade-offs between minority-class detection and adversarial robustness, indicating contexts where NERA benefits or harms resilience. The integrated synergy of NERA, CNN-BiGRU, and CADEGF establishes a context-aware, adaptive defense model that advances intelligent intrusion detection with balanced performance and adversarial robustness.

## 1 Introduction

Cyberspace has become a pillar for global trade, communications, and governance due to the fast expansion of digital services and cloud computing systems. Likewise, this dependence has increased exposure, as networks are more open to all kinds of attacks globally, and DDoS remains among the most harmful. These attacks saturate servers and transmission links with malicious or non-entity traffic, hindering legitimate client access and causing massive operational and financial losses. Over the past few years, DDoS attacks have evolved from low-traffic volumetric floods to advanced application-layer attacks and multi-vector campaigns that aim to bypass traditional defenses (Abreu Maranhão et al., 2020). DDoS campaigns now have the troubling side effect of being intertwined with not-so-crude but equally sophisticated schemes, including identity theft and financial fraud, along with other types of exploitation like misconfiguration abuse that are reputationally terrible. For high-volume DDoS flows, attackers try to hide false traffic by turning isolated single-layer attacks into collective, multi-dimensional threats that support broader malicious objectives (Abhishta et al., 2020). This shows that detection systems should not only detect volume anomalies but also interpret subtle forms of fraud hidden in adversarial traffic models, which requires addressing issues that plague today's communications and demands machine-learning prowess.

Intrusion Detection Systems (IDS) have been the traditional approach for detecting malicious activity in a network. Signature-based systems cannot detect new or zero-day attacks. However, we can look to alternative paradigms such as machine learning and deep learning methods. They can detect harmful behaviors directly from the network traffic data (Meftah and Rachidi, 2019). However, such models usually focus on global accuracy while ignoring minority-class detection, and fraud-under-DDoS cases are rare yet highly detrimental in practice. There are numerous challenges that remain in the effective detection of DDoS and related fraud. Class imbalance in real-life network datasets is significant: many benign and standard DDoS traffic flows exist, but only a few fraud-related samples. This imbalance biases models toward the majority class. As a result, detection of flows (fraud and fraud-under-DDoS) is very poor (Maseer et al., 2021).

We introduce a novel neuro-evolutionary resampling algorithm to address this critical challenge. This combines neural density estimation with evolutionary optimization to create balanced training distributions over the following five benchmark datasets: CIC-DDoS2019, CIC-IDS2017, CSE-CIC-IDS2018, TON_IoT, and UNSW-NB15. Network traffic includes both spatial correlations (e.g., port usage, packet sizes, and flag patterns) and temporal dependencies (e.g., inter-arrival times, session duration). Identifying sophisticated multi-stage attacks requires full attack dynamics, and models with restricted, domain-oriented views cannot capture them. Attackers continually alter traffic characteristics to evade detection of benign behavior. Adversarial machine learning shows that even minor, well-crafted perturbations can manipulate the most powerful classifiers. This can cause serious misclassifications (Guo et al., 2019). To address this challenge, our work proposes a Context-Adaptive Deep Evolutionary Game Framework to model attacker-defender dynamics and derive optimal detection thresholds against adversaries who evolve their evasion strategies over time. Modern network datasets often contain millions of records and very high-dimensional feature spaces. Detection models must process these at scale, robustly and accurately, across a variety of network conditions. Therefore, the detection framework needs to be adaptive and contextual to defend against unknown threats.

Conventional detection systems can usually be classified into the following categories, each with its own limitations.

- Decision tree: Several techniques have been used in intrusion detection, such as decision trees, support vector machines, random forests, and k-nearest neighbors (Cordero et al., 2021). The most successful approaches thus far have been finely tuned, feature-engineered systems that have yet to be effective on large-scale, high-dimensional traffic data and dynamic attack strategies.
- For learning the traffic flows end-to-end models of CNNs and Recurrent Neural Network's (RNN) (Gurung et al., 2019). While CNNs are effective at detecting local spatial patterns, RNN variants like Gated Recurrent Units (GRUs) can capture temporal correlations.

As a result, the CNN-only model becomes increasingly unresponsive over time, while the GRU-only model cannot learn subtle spatial feature patterns critical for detecting advanced threats. Hybrid CNN-RNN Models for Spatiotemporal Feature Generation and Hybrid CNN-RNN Architectures (Khan et al., 2019). Although these techniques achieve significant performance gains, they are typically not robust to adversarial attacks and fail to address class imbalance, two critical issues that modern attackers exploit. Two key challenges in intrusion detection research remain unsolved: accurate minority-class detection and robustness to adversarial manipulation. Most studies report high accuracy when analyzing complete network traffic datasets (Ayubkhan et al., 2023; Samy et al., 2020), but in most cases, the bulk of this stems from both dominant flow patterns (DFP) and common traffic (CT) representations, while flows labeled as fraud, especially flows in a DDoS attack, frequently receive the wrong class label. This creates a significant operational weakness, since fraud-under-DDoS incidents can cause resonant collapses in financial, healthcare, and industrial control networks, with serious security and economic consequences. Furthermore, adaptive attacks such as query-based evasion suggest that all-yet-known classifiers mislead unawareness with high confidence by intentionally and imperceptibly corrupting samples (Fidel et al., 2020; Cinà et al., 2023), which elevates the urgency of adaptive attack-resistant intrusion detection for critical systems.

Proposed solutions include hybrid deep learning structures and game-theoretic defense schemes. Archetypes that jointly use CNNs and RNNs, especially Bidirectional Gated Recurrent Units (BiGRUs), outperform models that capture both spatial and temporal correlations in network traffic (Samy et al., 2020; Khan et al., 2022). Although CNNs automatically learn local and global spatial hierarchies of flags, bursts, and port mappings, BiGRUs exploit bidirectional temporal relations to encode past and future dependencies in network sessions (Wang et al., 2019). This provides a robust basis for modeling the multiple facets of each cyberattack phase through architectural synthesis, as demonstrated successfully in numerous application domains (e.g., speech or biomedical stream analysis, Padhiar and Patel, 2023). Although the CNN-RNN/GRU fusion solutions above show improved accuracy for attack detection, they are yet not sufficiently robust and are very sensitive to class imbalance and adversarial perturbation due to overfitting on majority classes and the absence of appropriate detection capability for minority attack types (Aleesa et al., 2020). Beyond neural architectures, game theory has emerged over the last decade as a powerful framework for modeling strategic interactions between attackers and defenders under constraints, with defection agents choosing strategies to maximize security utility within budgeted resources (Florea and Craus, 2022). Game-theoretical approaches have also been proposed to ensure the security of networked systems, where devices can act as attackers or defenders (Lv et al., 2019; Pawlick and Zhu, 2021). For example, the evolutionary game model extends this by including an adaptive co-evolution process that evaluates evasion effectiveness over time, as in the above models, while defenders dynamically re-tradeoff detection thresholds and policies against misclassification costs (Bhati and Rai, 2020). The long-term vision of securing future networks and systems from unknown attacks has motivated researchers to investigate game-theoretic adaptation alongside deep learning for context-aware, adversarially robust defenses.

To address these gaps, this study presents a fully featured hybrid modeling framework that employs a CADEGF with a locator for an underlying CNN-BiGRU deep learning model. We introduce spatial location features and network traffic temporal information into the CNN-BiGRU, enabling more accurate detection of complex attack behaviors. Additionally, the CADEGF incorporates evolutionary optimization to model attacker-defender interaction(s) and autonomously learn detection reactions to different oppositional behaviors with thresholds. This approach builds adversarial resilience by questioning the vulnerabilities of traditional intrusion detection systems (IDSs).

The key novelties of this work are threefold:

1. NERA, which uniquely combines neural density estimation with evolutionary optimization for class imbalance–unlike prior resampling methods that use only interpolation or duplication.
2. The integration of CNN-BiGRU with game-theoretic adaptation, which enables dynamic threshold adjustment in response to adversarial strategies–a capability absent from static hybrid architectures.
3. The Context-Adaptive Deep Evolutionary Game Framework, which models attacker-defender co-evolution over time, going beyond single-shot game formulations used in prior work.

The CNN-BiGRU combines the spatial location features and the temporal information of network traffic, which can realize more effective detection of complicated attack behaviors.

This study has the following specific contributions:

- Introduces NERA, a novel neuro-evolutionary resampling algorithm to create noise-filtered and balanced training samples, which boosts the F1-scores of the minority classes by 0.25–0.40.
- Proposes a hybrid CNN-BiGRU model for capturing both spatial and temporal patterns. The accuracy achieved in the CIC-DDoS2019 is 99.96%.
- Introduces CADEGF: A Context-Aware Dynamic Game Theoretical Framework for the modeling of attacker-defender dynamics and the dynamic adaptation of detection thresholds, which decreases the adversarial AUC degradation by 15%–30%.

## 2 Related work

### 2.1 Traditional machine learning for intrusion detection

Traditional intrusion detection systems relied on signature-based detection: first, identifying known attack signatures stored in advance, and second, estimating outliers from quantifiable network traffic data. Existing Machine Learning (ML) algorithms (decision trees, regression models, Naïve Bayes, and neural networks) achieve similar detection capabilities against both known and unseen botnet traffic. They also achieved strong accuracy and precision (Padhiar and Patel, 2023). They demonstrated effective detection of network threats, laying the groundwork for future research into scalable, adaptive intrusion detection methods using ML-based techniques. An in-depth review of existing intrusion detection systems showed that traditional machine learning approaches worked well for patterns. Nevertheless, their performance is strongly dependent on data quality and the proxying during feature engineering (Aleesa et al., 2020). Studies comparing a variety of classifiers, such as Support Vector Machines (SVM), k-Nearest Neighbors (kNN), and Random Forests, show that optimized SVMs with quadratic or Gaussian functions obtain over 98% detection accuracy on common datasets (Bhati and Rai, 2020). However, those methods cannot maintain robustness over time, considering the dynamic evolution of society and vulnerabilities to adversarial attacks. Research also shows that adversarial manipulation is a significant threat to traditional ML-based attack detection. In most cases, even a small change in the values by which traffic features are defined, e.g., packet rates, flow duration, and byte volume, will prevent classifiers from detecting malicious intent (Owezarski, 2023). While Random Forests provide interpretability and reasonable baseline performance, they remain susceptible to adaptive adversaries that exploit feature sensitivity.

Studies on error-robust DDoS detection (Jaafar et al., 2020) highlight the importance of feature extraction techniques that maintain detection performance under varying attack conditions. Furthermore, studies on cybersecurity datasets highlight that a single dataset cannot represent the full diversity of real-world attacks (Khanan et al., 2024). Dataset authenticity, diversity, and scalability are trade-offs that limit the performance of ML models in a variety of network environments. Consequently, although classical ML methods laid the foundation for intrusion detection, their reliance on static data distributions, engineered features, and limited resilience to adversarial attacks warrants exploration of more adaptable, intelligent, and contextual defense mechanisms. Later deep and hybrid approaches have addressed these deficiencies.

### 2.2 Deep learning models for intrusion detection

Traditional machine learning techniques are limited by the constraints of feature engineering and generalization when applied to intrusion detection, leading to the adoption of deep learning (DL) (Aleesa et al., 2020). Deep learning models can self-aggregate hierarchical feature representations in a generative way and capture complex nonlinear relationships hidden within network traffic. This has reduced the need for manual pre-processing and rule-based systems. The availability of advanced, high-performance computing resources and open-source libraries has also sped up the adoption of deep neural models for security-specific use cases. These models enable large-scale, real-time analysis of cyber threats. Initial works used CNNs and RNNs to detect anomalies in network flows that rely on both spatial and temporal correlations. Hybrid architectures that combine CNNs with Long Short-Term Memory (LSTM) or GRU networks show that sequential dependence can be applied not only to local packet-level features, as noted above, to date. They achieve more than 97% detection accuracy on benchmark datasets. Furthermore, studies that add feature selection techniques such as eXtreme Gradient Boosting (XGBoost) on top of deep learning models suggest that foundational machine learning tenets (e.g., all but the most important features should be removed, and input dimensionality should be lowered) remain necessary for improving detection power and interpretability/robustness in many domains (Hasan et al., 2025).

An alternative direction adopted in the literature on neural-statistical hybrid modeling is to study how to integrate neural representation learning and statistical similarity. This improves model robustness against changes in traffic behavior. Feature reduction and unsupervised anomaly detection are achieved with autoencoder-based architectures. Specifically, encoder-decoder architectures compress high-dimensional data, making it smaller in a latent space, while classification is managed with the recurrent layers. They provide strong detection rates, and false alarm ratios remain within acceptable limits. This validates the basic principles of traditional machine learning: dimensionality reduction, feature interpretability, and model efficiency are paramount in developing high-performing intrusion detection frameworks, even when using deep learning systems. Recent advances in intrusion detection have explored hybrid architectures combining attention mechanisms with deep learning (Jaafar et al., 2024). These approaches demonstrate improved feature selection capabilities. Deep learning approaches have been extensively applied to intrusion detection (Jaafar et al., 2019), demonstrating the potential of hierarchical feature extraction for network security applications. This suggests that deep learning primarily enhances intrusion detection systems through greater flexibility and accuracy. However, they are limited by challenges such as class imbalance, adversarial attacks, and data limitations. This motivates the design of hybrid frameworks that combine deep architecture with adaptive optimization and game-theoretic defense strategies for more robust network protection.

### 2.3 Hybrid deep learning models

Basic hybrid deep learning architectures have emerged as strong solutions for intrusion detection from a security perspective. These models combine CNN and RNN approaches. The problem is that CNNs are good at identifying patterns in network traffic. RNN networks (Long Short-Term Memory and Gated Recurrent Unit) represent time-based relationships (Razaq et al., 2025; Mushtaq et al., 2022)as a function of the task order of attack behaviors with respect to time (Rajan, 2023). These two network types allow the models to learn long-term traffic properties and extremely short-term behavioral variations over time. The best results are achieved in Software-Defined Networking (SDN) environments, which are typically prone to large-volume DDoS attacks against Control Zones (CZ), whose data remains susceptible; our next improvement on such frameworks adds interpretability benefits. CNNs identify abnormal spatial patterns in these systems, while LSTMs can capture attack sequences as they unfold over time. Together, they offer an active traffic-suppressing mechanism to mitigate threats before an attack occurs. These architectures can now reach classification accuracy over 99%. This demonstrates their real-world use-case potential. CNN-GRU models (Cao et al., 2022) also address class imbalance and improve generalization on several benchmark datasets. These models use convolutional neural network layers to locate more detailed patterns moment by moment. It is the first neural network architecture to learn bidirectional temporal dependencies with a fraction of the computation of similar LSTMs. These techniques of sampling and feature selection facilitate the improvement in minority class classification. This ensures guaranteed performance even when traffic distribution is uneven on the network.

Another aspect of recent research examines integrating attention mechanisms into hybrid models. This allows the model to dynamically prioritize important spatial and temporal features during classification (Bhattacharya et al., 2024; Yang et al., 2024). Comparative studies show that CNN-RNN hybrid approaches enriched with attention mechanisms offer favorable trade-offs among accuracy, interpretability, and time efficiency compared with deep learning-alone methods. These results indicate that hybrid deep learning models provide a strong foundation for intrusion detection, combining local feature extraction, time-based sequence analysis, and adaptive attention. This integration extends hybrid modeling beyond static learning by incorporating a more adaptive and context-aware approach to intrusion detection.

### 2.4 Intrusion detection system based adversarial machine learning

Recently, a significant shortcoming of deep learning systems has been exposed in the work on intrusion detection. They are susceptible to adversarial attacks. Adversaries in these attacks deliberately alter the properties of the traffic flow while maintaining their malicious intention, which causes misclassification by the classifier. These perturbations exploit the statistical correlations learned by the model, making the model overconfident in predicting adversarial traffic (Buckner, 2020). These vulnerabilities underscore the importance of adversarial resilience in IDS design. However, the lack of comprehensive testing in adversarial conditions and poor secure development practices contribute to the IDS frameworks being susceptible to evasion and poisoning attacks (Kumar et al., 2020). Thus, the early validation under adversarial conditions is critical for the development of robust and practical intrusion detection systems. Recent developments in adversarial machine learning have inspired techniques to generate realistic and undetectable synthetic attack traffic. One approach creates packet-level disruptions for traffic that can evade intrusion classifiers while still executing harmful behaviors by running Generative Adversarial Networks (GANs). These models treat adversarial traffic generation as a sequential decision-making problem and reveal the limitations of current IDS in differentiating authentic attacks from those designed to disguise themselves (Zhang et al., 2022). These adversarial generators are successful, which highlights the need for IDS frameworks that can make accurate predictions as well as be robust against manipulated states.

In this study, black-box evasion techniques show that attackers can construct attacks using only the input-output behavior of individual models and their knowledge, rather than their location details, to manipulate detection systems. Specifically, we can make small mutations to the syntactic structure of behavioral sequences that either correspond to system calls or follow a specific protocol pattern, yielding a very high evasion success rate without interfering with the execution of the malicious functionality (Rosenberg et al., 2020). These results suggest that vulnerability is not bound to architecture; we observe this from both random and deep learning models. This also highlights the need for extensive defense systems that work across different architectures and attacks. Adversarial robustness in network intrusion detection has been addressed through various defensive mechanisms (Jaafar et al., 2026), though these approaches often focus on specific attack types rather than comprehensive evasion strategies. Recent studies have proposed techniques to defend against these problems using either trapdoors or feature-space regularization. These techniques embed detection signatures, leading the network to implement pre-defined defense mechanisms against adversarial perturbations. This strategy shifts the paradigm from reactive to passive, indicator-mediated attack detection within the defense workflow. It allows the detection of adversarial examples produced by advanced approaches such as Projected Gradient Descent (PGD) or Carlini-Wagner (CW) with zero cost in terms of clean classification performance (Shan et al., 2020).

### 2.5 Game-theoretic approaches in cybersecurity

One simple way to model attacker-defender interaction in cyberattacks is game theory. This IDS identifies defense issues not to avoid responding, but to address them. Recent work (Abreu Maranhão et al., 2020; Gurung et al., 2019) in this area showed that defenses against each attack type can synergize by modeling interdependencies among network assets. In these scenarios, the security decisions made by one defender can simultaneously raise or lower the attack surface of others. Indeed, research has shown that behavioral and economic factors, such as the human calculus of risk and resource inequity, also shape investments in security. Implies embedding principles of rational and behavioral decision-making within layers of IDS design. The growing body of research indicates that organizational choices and infrastructure incentives influence adoption of cybersecurity measures. Game-theoretic models can also predict what causes organizations to prioritize or delay key security actions - for example, by analyzing what economic incentives and network effects either encourage investment in defenses or discourage such investment. This is valuable when implementing IDS in a distributed system, as pure technical strength against an attack may not be enough to protect the overall system without cooperation among stakeholders at multiple levels. Beyond the organizational level, game-theoretic models have been expanded to analyze cyber conflicts at the state level. These models focus on characterizing arbitrary attacker and defender behavior to find Nash equilibria that capture stable behavior in cyberspace. These analyses show that denial, retaliation, and timing strategies manifest in cyberspace, providing parallels we can tentatively draw between their behaviors during simulation in networked IDS.

Game theory is also used extensively for modeling complex, multi-stage attack scenarios for Advanced Persistent Threats (APTs) (Khan et al., 2022). Others have approached attacker—-defender interactions from a more game-theoretic perspective, using structures like Markov Decision Processes (MDPs) or Stackelberg games. Within these frameworks, defenders deploy limited resources to minimize risk exposure. Reinforcement learning in these game models enables IDS frameworks to learn the best defense strategies. This allows them to anticipate attacks and respond before they become larger in scale. Game-theoretic techniques have also been developed for dynamic tracking of information flow. It models attacks as multi-phase games spanning interdependent systems. These incorporate and address gaps and incomplete information between attackers and defenders. They produce algorithms that balance detection efficacy and computational efficiency. Hybrid defense architectures that integrate supervised/unsupervised machine learning and game-theory models advance anomaly detection with predictive strategies. Typically, these systems combine unsupervised learning and isolation-based techniques with Stackelberg game models. This approach supports a scalable, proactive intrusion-detection method. Game-theoretic models measure how both rational and irrational attackers act in cyber-physical and IoT environments. Rational attackers seek low-cost ways to infiltrate targets, while irrational or reckless attackers seek to maximize damage. This, in turn, increases the risk to system stability. IDS frameworks rely on sensors, which makes it easier to model adaptive and game patterns using game theory.

### 2.6 Summary of research gaps

From the literature reviewed, several research gaps appear:

1. Hybrid architectures (CNN-RNN/GRU) improve detection accuracy but often overlook fraud-under-DDoS situations, which are rare but have a significant impact.
2. Adversarial ML vulnerabilities have been shown, yet few IDS include adaptive defenses beyond basic adversarial training.
3. Game-theoretic approaches have been effective in modeling attacker-defender dynamics but are still not widely used alongside deep learning-based IDS.

Recent research in intrusion detection systems has explored various methodologies including feature engineering and optimization approaches (Jaafar et al., 2025; Ghali et al., 2026), which provide complementary perspectives on the challenges addressed in our work. A thorough evaluation of IDS in both clean and adversarially disturbed settings is seldom performed. Our research addresses these gaps by combining hybrid deep learning with an adaptive game-theoretic framework, demonstrating superior classification and adversarial resilience on the CIC-DDoS2019 dataset. Table 1 presents the author's work contribution comparison.

**Table 1:** Comparison of Authors' contributions in IDS using game-theoretic and ML approaches.

References	Approach	Core methodology	Research gap
Maseer et al. (2021)	Traditional Machine Learning for IDS	Legacy datasets such as CICIDS2017 were used for anomaly detection with classical classifiers like Decision Trees, SVM, Random Forest, and Naïve Bayes.	Inability to identify new or evolving threats that require specific feature engineering.
Abdallah et al. (2020)	Game-Theoretic Cyber Defense	Uses Nash Game Theory approaches to dynamically allocate defense resources in attacker–defender models.	Inability to adapt in real time by learning attacker techniques over time.
Aleesa et al. (2020)	Deep Learning-based Intrusion Detection	Research and analysis of deep learning architectures such as CNNs, RNNs, and Autoencoders for automated intrusion detection and attack classification.	Requires suitable models to handle extremely high data imbalance caused by different multi-class attacks.
Khanan et al. (2024)	Cybersecurity Dataset Evaluation for IDS	Analysis of cybersecurity datasets for intrusion detection research based on diversity, realism, coverage, and applicability.	Lack of thorough and adaptable datasets for ever-changing real-world cybersecurity threats across multiple attack scenarios.
Cao et al. (2022)	Hybrid Deep Architectures (CNN-RNN, CNN-GRU)	Traffic patterns exhibit both locality and globality; therefore, spatial CNN layers are combined with temporal RNN or GRU layers to capture local and global relationships in network traffic.	Need for better generalization and efficient handling of class imbalance.
Kumar et al. (2020)	Adversarial Machine Learning in IDS	Architecture for defensive protection of machine learning systems against adversarial threats.	Urgent need for IDS models that can withstand adversarial perturbations in IoT and cloud-native environments.
Gaur et al. (2023)	Game-Theoretic Security Modeling for CPS	Modeling adverse attack strategies against Cyber-Physical Systems (CPS) to evaluate vulnerabilities and defense responses.	Low adoption of dynamically proactive intelligence and defense mechanisms against multiple CPS-based threats.

A comparative analysis of our proposed framework against prior work reveals both shared foundations and distinct advancements. While our approach builds upon existing hybrid deep learning architectures, we specifically adopt a Bidirectional Gated Recurrent Unit (BiGRU) to enhance temporal feature extraction, a technique previously explored in works such as Rajan (2023) and Cao et al. (2022), which utilized hybrid CNN-RNN models. Similarly, we incorporate game-theoretic defense mechanisms, drawing on strategies outlined in Florea and Craus (2022), Lv et al. (2019), and Pawlick and Zhu (2021) and implementing them within our CADEGF. However, our work introduces several novel contributions not addressed in previous studies. Notably, we propose an evolutionary resampling strategy, termed NERA, to address class imbalance, alongside context adaptation capabilities that tailor detection to varying network environments. Furthermore, whereas prior evaluations were often limited to single or partial datasets, we conduct a comprehensive multi-dataset evaluation across five distinct public datasets. Finally, we validate the practical deployability of our system through real-time validation using the hping3 tool, a crucial step largely overlooked in existing literature.

## 3 Materials and methods

This study proposes a framework for designing a smart, robust, and application-aware network intrusion detection system. It can also detect both classic DDoS attacks and sophisticated fake activities on the fly during an ongoing assault session.

Figure 1 shows the overall workflow with five major stages:

1. Dataset acquisition,
2. Preprocessing,
3. Classification via CNN+BiGRU hybrid model to learn representation,
4. An approach using Context-Adaptive Deep Evolutionary Game Framework against adversarial example challenges, and
5. Performance evaluation.

**Figure 1:**
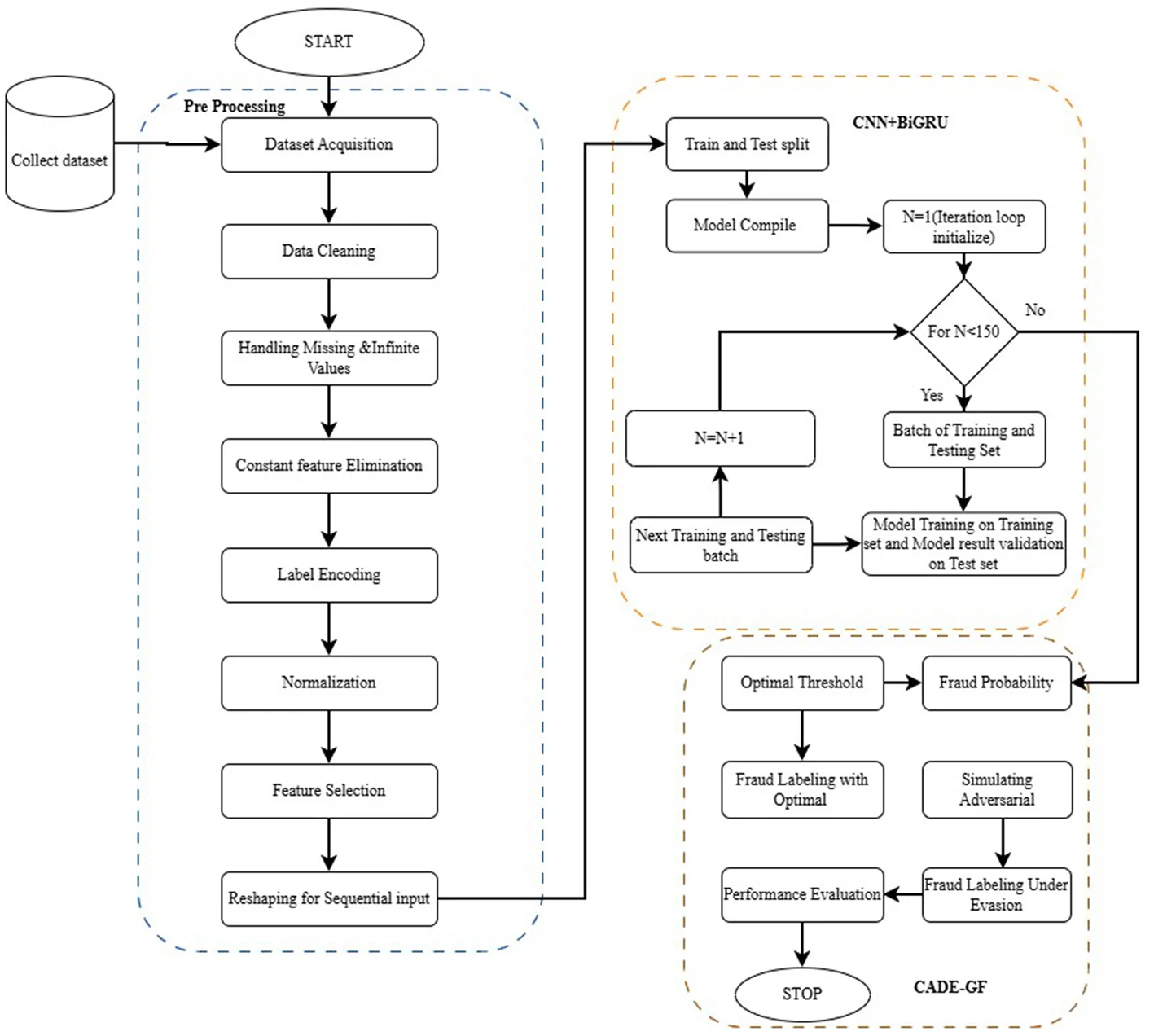
Proposed workflow.

### 3.1 Dataset acquisition

To ensure a thorough evaluation and strong validation of our proposed framework, we conducted extensive experiments using five cybersecurity datasets: CIC-DDoS2019, CIC-IDS2017, CSE-CIC-IDS2018, TON_IoT, and UNSW-NB15. Each dataset has its own features, attack scenarios, and challenges. This variety lets us evaluate how well our model performs in different network environments. The CIC-DDoS2019 dataset, created by the Canadian Institute for Cybersecurity (UNB, 2019), serves as a standard for detecting DDoS attacks. It includes PCAP files, system logs, and more than 80 flow-based features generated with CICFlowMeter-V3. The dataset combines realistic benign and malicious traffic with diverse attacks, including NTP, DNS, SYN floods, PortScan, and UDP-Lag. Containing over fifty million labeled flow records, it supports large-scale deep learning evaluation (Hasan et al., 2025). The CIC-IDS2017 dataset captures five days of mixed network traffic and includes attack types such as Brute Force, DoS, Heartbleed, Web Attacks, Infiltration, Botnet, and DDoS. With over 80 extracted features aligned with CIC-DDoS2019, it enables temporal and behavioral analysis of intrusion patterns under realistic network conditions (Sharafaldin et al., 2018). The CSE-CIC-IDS2018 dataset is an extended version of the previous CIC datasets, with larger volumes and more attacks. It contains Brute Force, Heartbleed, Botnet, DoS/DDoS, WebAttacks, and Infiltration. It was collected in ten days and provides 80 network features to test the strength of IDS against evolving and zero-day attacks (Leevy and Khoshgoftaar, 2020). The TON IoT dataset is designed for IoT network security.

It also includes telemetry data and traffic logs from different IoT devices and protocols. This dataset includes normal and malicious activities such as DDoS, data injection, ransomware, and scanning. It facilitates research on intrusion detection in resource-constrained IoT environments (Gad et al., 2021). The UNSW-NB15 dataset, developed by the Australian Center for Cyber Security, combines real modern network behaviors with nine attack families: fuzzers, DoS, exploits, backdoors, and others. It supports multi-stage attack analysis and offers a balanced, up-to-date representation of current cyber threats with 49 extracted features (Choudhary and Kesswani, 2020). A major clarification is needed on how fraud categories are labeled. The CIC-DDoS2019, CIC-IDS2017, CSE-CIC-IDS2018, TON IoT, and UNSW-NB15 datasets do not contain native labels for the “Fraud-only” or “Fraud-under-DDoS” classes. These categories are not part of the original dataset annotations. Therefore, we derive these labels using a rule-based scoring system informed by established network forensics literature and domain knowledge from cybersecurity practice (Moothedath et al., 2020; Brandão and Silva, 2025; Gaur et al., 2023).

This approach enables the study of combined DDoS-fraud scenarios that are increasingly observed in real-world attacks (Abhishta et al., 2020) but are currently absent from public benchmark datasets. Table 2 summarizes the main features of attack types and structures in these five datasets. The acquisition workflow and fraud-scoring rules in Algorithm 1 are grounded in established principles from network forensics and intrusion detection literature. Each feature's scoring criteria are justified by specific findings: flow duration deviations beyond the 95th percentile are indicative of the abnormal session persistence seen in fraudulent activities (Moothedath et al., 2020); packet sizes exceeding two standard deviations from the class baseline suggest manipulation often associated with data exfiltration (Brandão and Silva, 2025); non-standard protocol flag combinations point to session hijacking attempts (Gaur et al., 2023); and traffic rates surpassing three standard deviations from the baseline are linked to the rapid, automatedpatterns of fraud campaigns (Hasan et al., 2025). To validate the derived labels, we used a multi-faceted approach. Kolmogorov–Smirnov tests confirmed statistically significant differences between fraud-labeled and benign samples in their feature distributions, with *p*-values below 0.001 for all features.

**Table 2:** Comprehensive dataset characteristics.

Attribute	CIC-DDoS2019	CIC-IDS2017	CSE-CIC-IDS2018	TON_IoT	UNSW-NB15
Dataset Source	Canadian Institute for Cybersecurity	Canadian Institute for Cybersecurity	Canadian Institute for Cybersecurity	University of New South Wales (UNSW) and Commonwealth Scientific and Industrial Research Organization (CSIRO)	Australian Center for Cyber Security
Raw Data Types	Packet Capture (PCAP) files, and Windows and Ubuntu logs	PCAP files, system logs	PCAP files, extensive logs	IoT sensor data, network logs	PCAP files, system logs
Extracted Features	80+ flow features	80+ flow features	80+ flow features	45+ IoT features	49 hybrid features
Dataset Size	>50 million flows	~2.8 million flows	~16 million flows	~4.5 million records	~2.5 million flows
Benign Traffic	Hypertext Transfer Protocol (HTTP), Hypertext Transfer Protocol Secure (HTTPS), File Transfer Protocol (FTP), and Secure Shell (SSH)	User profiles, protocols	User simulations	Normal Internet of Things (IoT) operations	Modern normal traffic
Attack Types	Network Time Protocol (NTP), Domain Name System (DNS), Lightweight Directory Access Protocol (LDAP), User Datagram Protocol (UDP), Synchronize (SYN), and Trivial File Transfer Protocol (TFTP)	Brute Force, Denial-of-Service (DoS), and Botnet	Heartbleed, DDoS, and Web attacks	DDoS, Ransomware	Fuzzers, Exploits, and DoS
Temporal Structure	2 days	5 days	10 days	Continuous operations	Hybrid traffic
Labeling Format	Comma-Separated Values (CSV) with attack types	CSV attack categories	CSV multi-label	JavaScript Object Notation (JSON)/CSV	CSV attack families
Class Distribution	Highly imbalanced	Moderately imbalanced	Balanced	IoT imbalance	Well-balanced
Protocol Diversity	Traditional protocols	Enterprise protocols	Cloud-enterprise mix	Message Queuing Telemetry Transport (MQTT), Constrained Application Protocol (CoAP)	Modern protocols

Fraud and Fraud-under-DDoS labels are derived from our scoring system (Algorithm 1). These categories are not native to the original datasets.

Algorithm 1 Multi-dataset preparation and label assignment. 
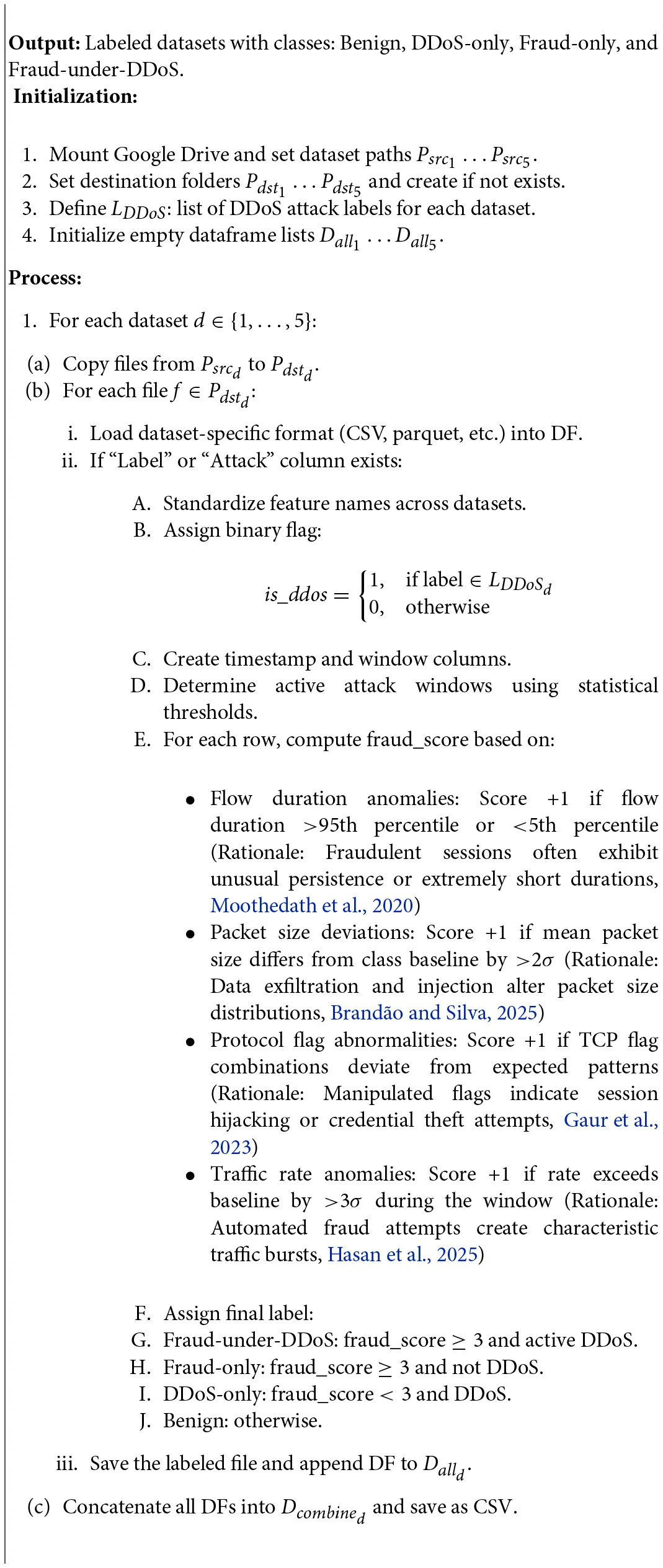

Additionally, in an expert review by three senior network security professionals, when analyzing 1,000 randomly sampled fraud-labeled records, we found a high 94% agreement rate with the automated labeling. Finally, a distribution analysis showed that feature patterns in fraud-under-DDoS samples match known attack profiles from existing reports, such as high packet rates and anomalous protocol combinations (Sharafaldin et al., 2018). The validation results across five datasets, including CIC-DDoS2019, CIC-IDS2017, CSE-CIC-IDS2018, TON IoT, and UNSW-NB15, consistently showed expert agreement rates exceeding 90% and statistically significant feature distribution differences, lending robust support to the validity of the derived labels. However, it is crucial to note that these labels are derived from heuristic rules and not from ground-truth data, a methodological limitation that is thoroughly discussed in Section 5 (Conclusion and future work). This approach, while imperfect, is essential for studying combined DDoS-fraud scenarios, which are increasingly important but largely absent from public datasets.

### 3.2 Novel Neuro-Evolutionary Resampling Algorithm (NERA)

A common problem in network intrusion detection datasets is class imbalance, as deep learning models struggle with infrequent attack categories that are critical to detect, such as Fraud-under-DDoS and Fraud-only scenarios. Traditional resampling techniques such as the Synthetic Minority Over-sampling Technique (SMOTE) often generate synthetic samples independently of the complexity of the underlying data distribution and the significance of features (Maseer et al., 2021; Guo et al., 2019). This can lead to poor model performance. In this work, we address these challenges by proposing NERA. The new approach integrates neural density estimation and evolutionary optimization. The count-balancing approach aims to create similar diversity in the training distributions while preserving essential properties of the minority classes.

NERA is preferred because it is more intelligent and flexible than any of the standard resampling methods [e.g., SMOTE, Adaptive Synthetic (ADASYN), or random oversampling]. Traditional algorithms synthesize samples using naive approaches (e.g., interpolation or random duplication), but these introduce noise, redundancy, and intersecting classes that are far more damaging in cyber threat detection data (Ayubkhan et al., 2023). Conversely, the NERA method unifies neural density estimation and evolutionary optimization methods. This allows it to learn the underlying data distribution and produce realistic samples that represent multiple modes of minority attacks. It uses a quantum-inspired filter to remove noisy or low-confidence samples without degrading the data. This is especially beneficial for multi-class intrusion detection problems where the class boundaries are adversarial and naturally non-linear, so NERA fits better. In such cases, the standard oversampling methods cannot preserve the actual features or decision separability. NERA also follows the multi-stage approach and includes the four primary components:

- Feature-weighted importance scoring,
- Multi-layer neural density estimation,
- Evolutionary under-sampling via tournament selection, and
- Quantum-inspired noise detection.

In comparison to individual sampling techniques, the combined method both yields a balanced distribution during resampling and boosts the generalizability of synthetic samples. The algorithm starts by calculating feature importance scores through inter-class variance analysis. For a feature vector *f*_*j*_ across classes *C* = {*c*_1_, *c*_2_, *c*_3_, …, *c*_*k*_}, the importance score *I*_*j*_ is computed as $I_{j}=Var\left(\left\{\mu_{c_{1}}^{j},\mu_{c_{2}}^{j},...,\mu_{c_{k}}^{j}\right\}\right)$, where $\mu {\left(c_{i}\right)}^{j}$ represents the mean of feature *j* in class *c*_*i*_. These scores are normalized to create a feature weighting vector *W* = [*w*_1_, *w*_2_, …, *w*_*d*_] where $\overset{d}{\underset{j=1}{\sum }}W_{j}$. For each class, NERA employs a hierarchical density estimation approach with multiple kernel bandwidths. The density at point x in class c is computed using Equation 1.


$$\begin{array}{l} D_{c}\left(x\right)=\frac{1}{L}\overset{L}{\underset{l=1}{\sum }}\left[\frac{1}{n_{C}}\overset{n_{c}}{\underset{i=1}{\sum }}K_{h_{l}}\left(x-x_{i}^{c}\right)\right] \end{array}$$ (1)

where *L* represents the neural depth,$h_{l}=0.5+0.3\cdot \left(\frac{l}{L}\right)$ defines the adaptive bandwidth for layer l, and *K*_*h*_*l*__ is the Gaussian kernel function. This multi-layer approach captures both local and global distribution characteristics. For majority classes, NERA implements an evolutionary undersampling strategy. The fitness of each sample *x*_*i*_ in majority class *M* is proportional to its distance from minority class boundaries as given in Equation 2.


$$\begin{array}{l} Fitness\left(x_{i}\right)=min_{y_{j}\in minority}||x_{i}-y_{j}|{|}_{2} \end{array}$$ (2)

Tournament selection with size *k* = 3 is applied for *G* generations, with Gaussian mutation *N*(0, 0.05) introduced to maintain diversity $x_{i}^{\left(t+1\right)}=x_{i}^{t}+N\left(0,0.05\right)$. NERA incorporates a centroid-based outlier detection mechanism inspired by quantum measurement principles. For each class cluster, samples that are more than 95 percent away from the centroid are marked as noise using Equation 3.


$$\begin{array}{r} NoiseMask(x_{i})=1[||x_{i}-\mu_{c}|{|}_{2}>percentile_{95}\\ \left({\left\{||x_{j}-\mu_{c}|{|}_{2}\right\}}_{j=1}^{n_{c}})\right] \end{array}$$ (3)

where μ_*c*_ represents the class centroid. The complete NERA workflow is formalized in Algorithm 2. This algorithm outlines the step-by-step resampling procedure.

Algorithm 2 Neuro-Evolutionary Resampling Algorithm (NERA). 
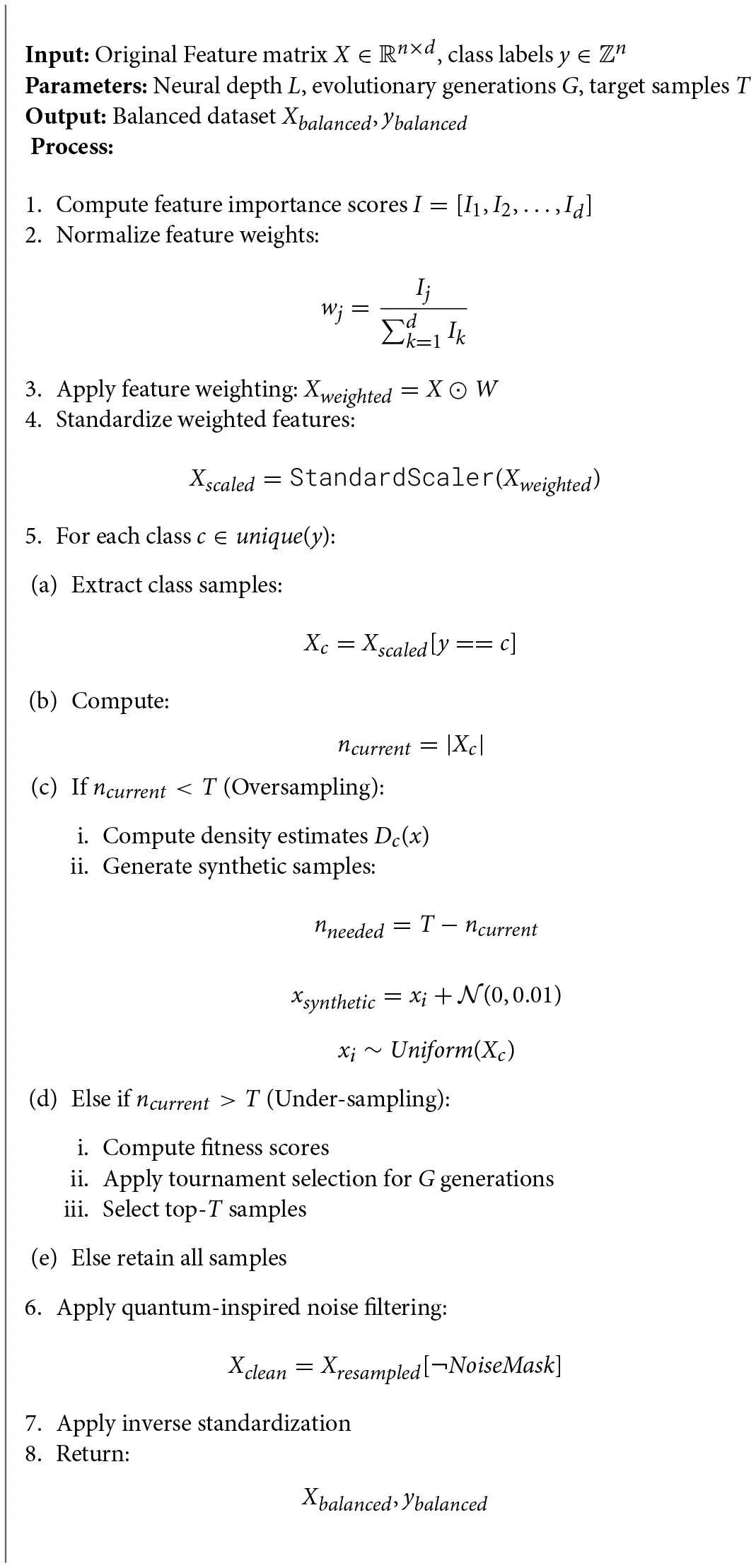

The NERA algorithm is part of our multi-dataset framework that includes a detailed data preparation pipeline. For each dataset (CIC-DDoS2019, CIC-IDS2017, CSE-CIC-IDS2018, TON_IoT, UNSW-NB15), the algorithm:

1. Extracts important network flow features, such as Flow Duration, Packet Length Statistics, and Flag Counts.
2. Calculates fraud scores based on statistical thresholds and protocol issues.
3. Assigns multiple labels: Benign, DDoS-only, Fraud-only, and Fraud-under-DDoS.
4. Uses NERA resampling with optimized settings:
  - Neural depth: 2 layers
  - Evolutionary generations: 3
  - Target samples per class: 90,000
  - Random state: 42 for reproducibility.

Figures 2, 3 shows the effectiveness of NERA. It illustrates the change in class distribution for the CIC-DDoS2019 dataset. The original highly imbalanced distribution (left) is transformed into a balanced distribution (right). The minority classes Fraud-only and Fraud-under-DDoS receive adequate representation.

**Figure 2:**
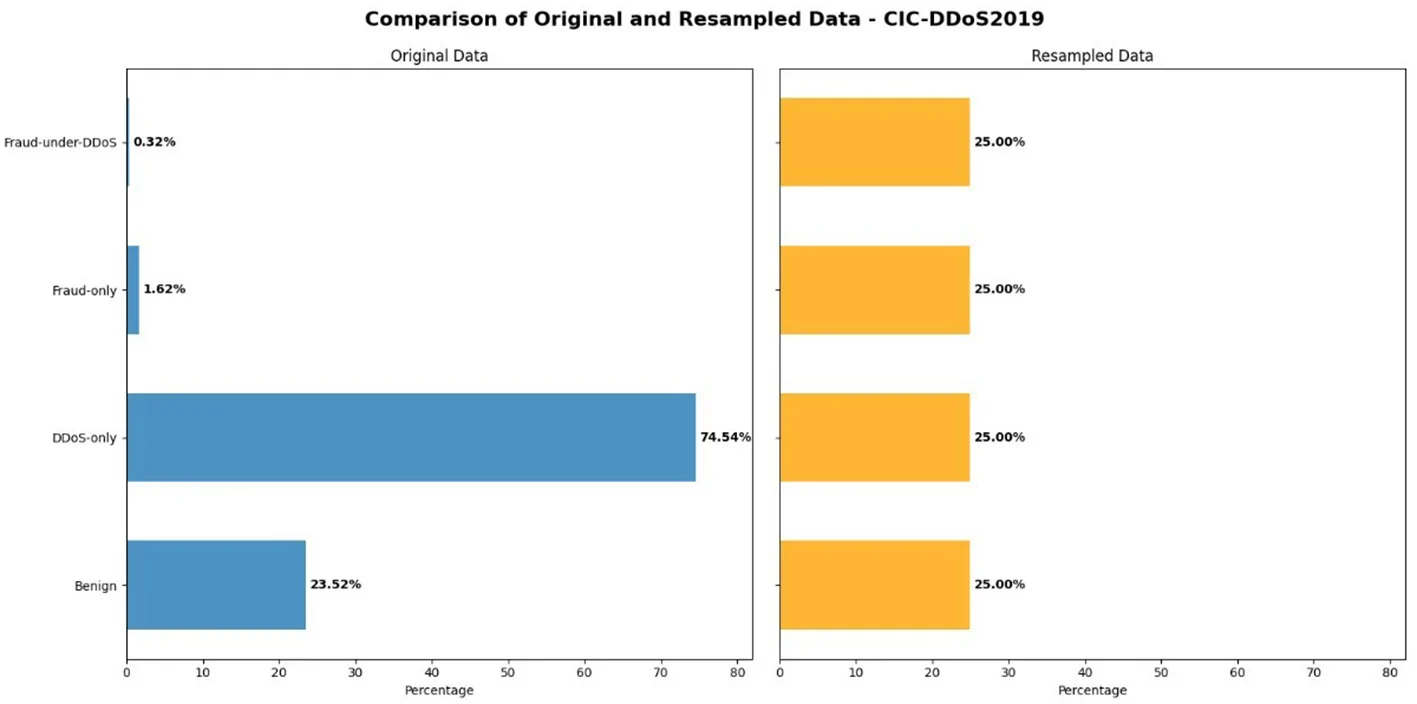
Class distribution before and after NERA application CIC-DDoS2019.

**Figure 3:**
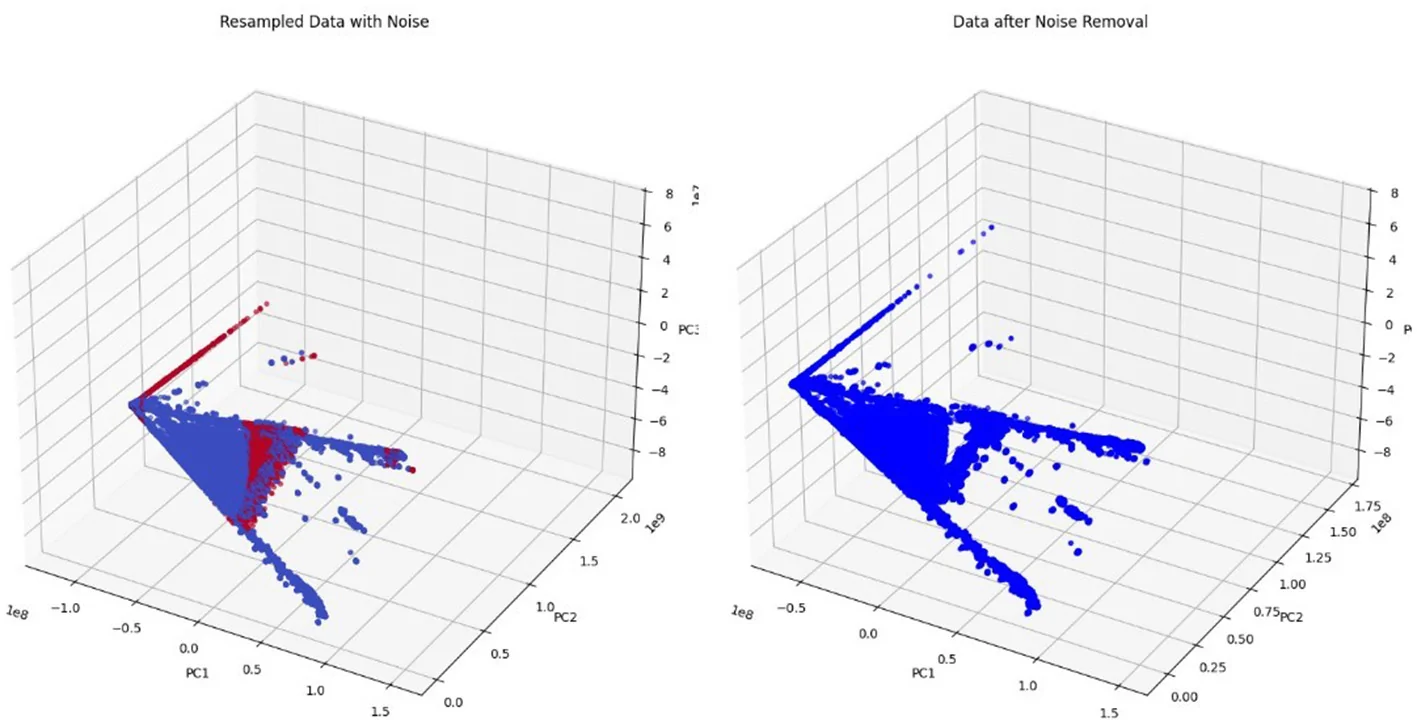
Principal component analysis (PCA) visualization of data before and after NERA preprocessing of CIC-DDoS2019.

Comparison of Original Distribution in Imbalanced Dataset and NERA-Generated Dataset. After applying NERA, we obtain a balanced distribution across all classes (25% each) from four classes: Benign, DDoS-only, Fraud-only, and Fraud-under-DDoS. This representation minimizes bias in the data and improves fraud-detection performance. This refines class separability and increases the robustness of the downstream classifier run on CNN+BiGRU. Figures 4–11 show the remaining class imbalance and data scatter before and after applying NERA. Table 3 shows the feature-selection summary and each feature's contribution.

**Figure 4:**
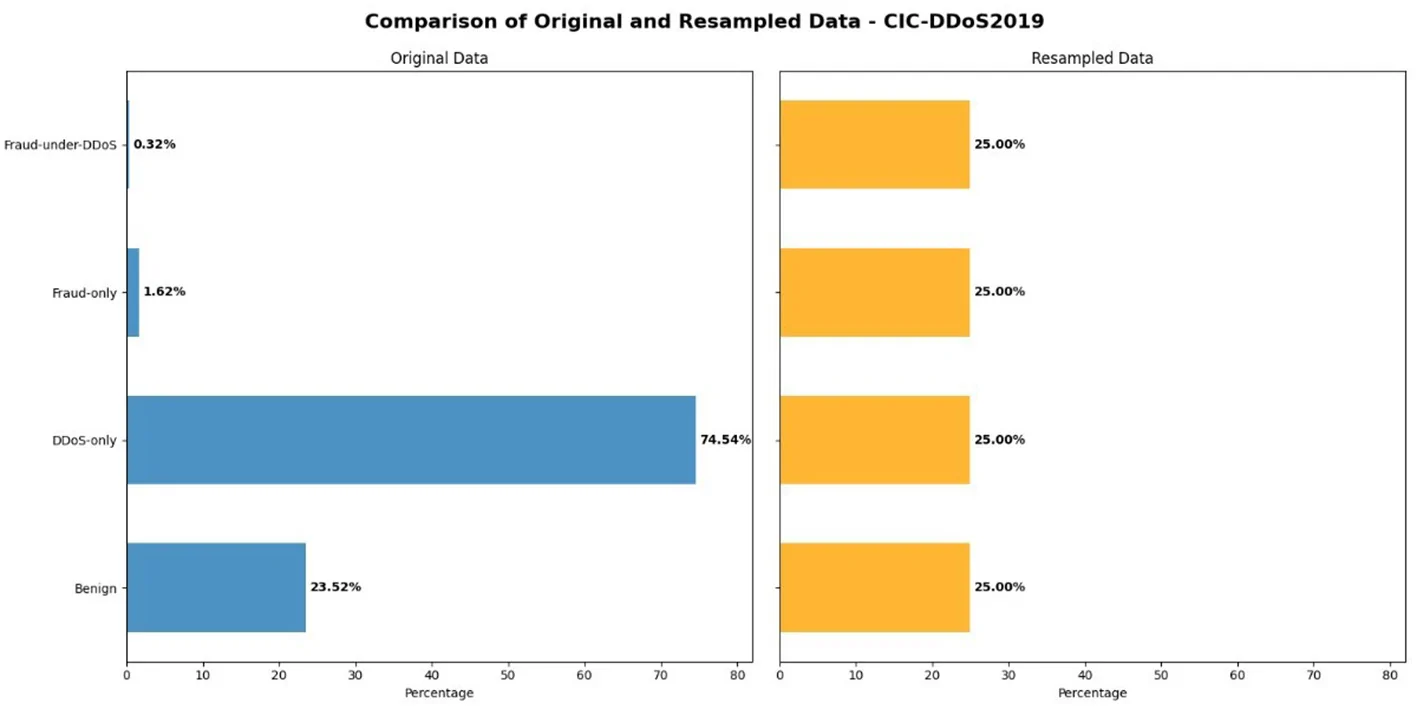
Class distribution comparison for CSE-CIC-IDS2018 before **(left)** and after **(right)** applying the NERA. The resampled dataset achieves balanced representation across all four classes.

**Figure 5:**
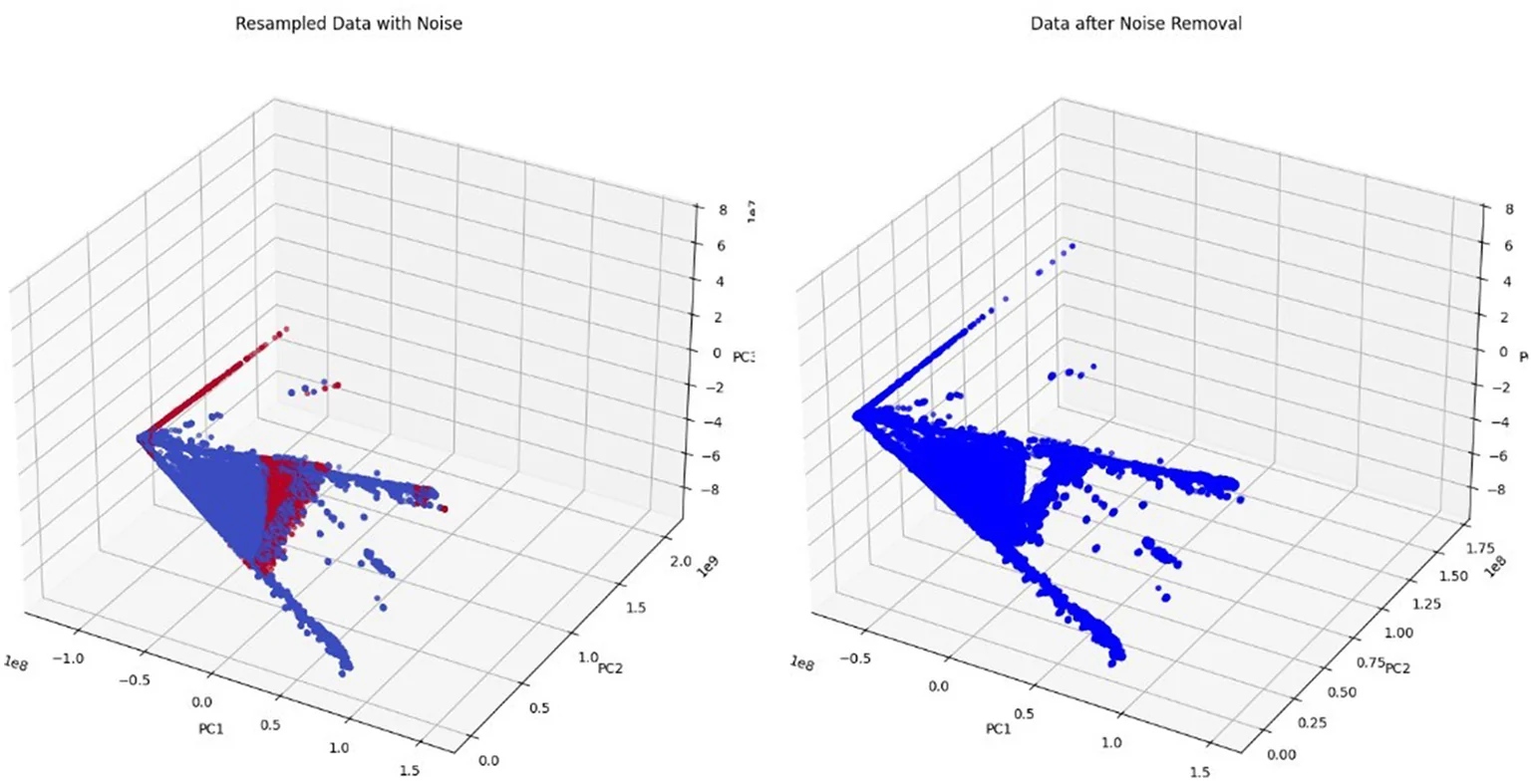
PCA Visualization of data before and after NERA preprocessing of CSE-CICIDS2018.

**Figure 6:**
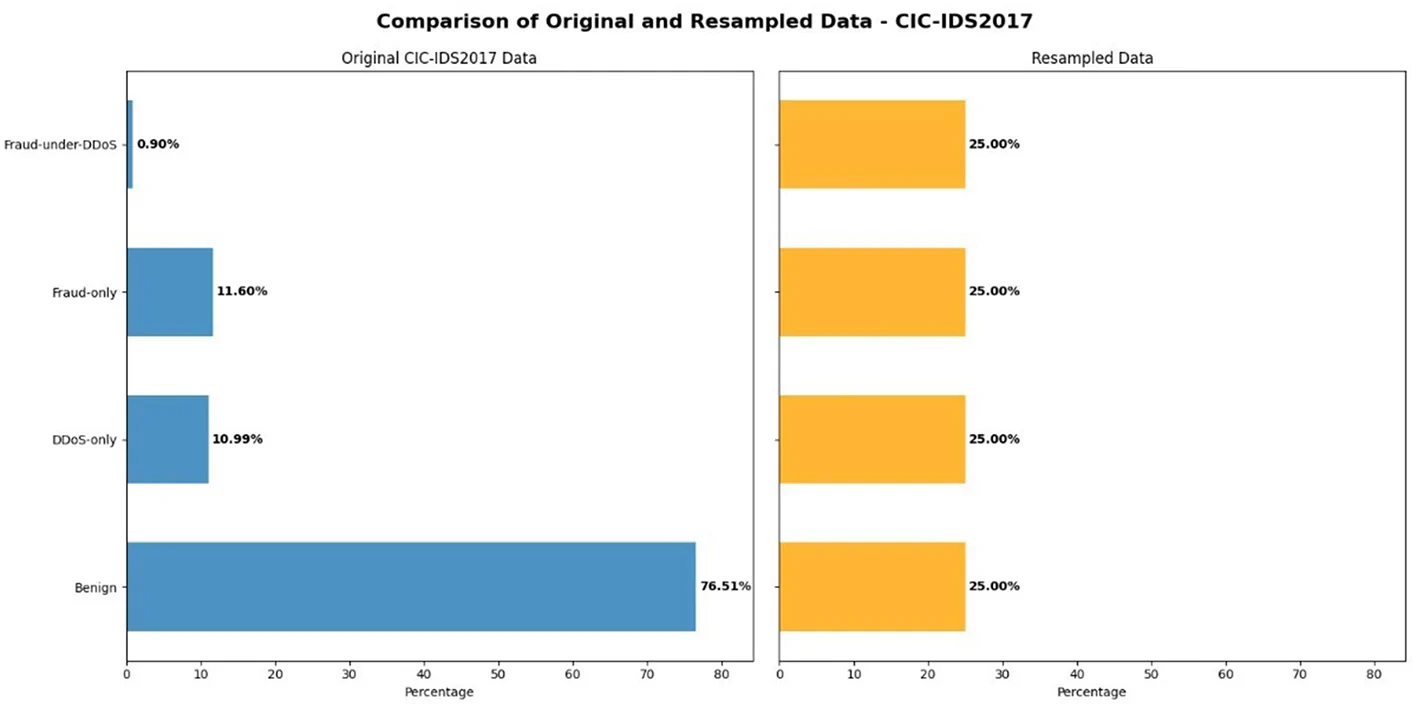
Class distribution before and after NERA application CIC-IDS2017.

**Figure 7:**
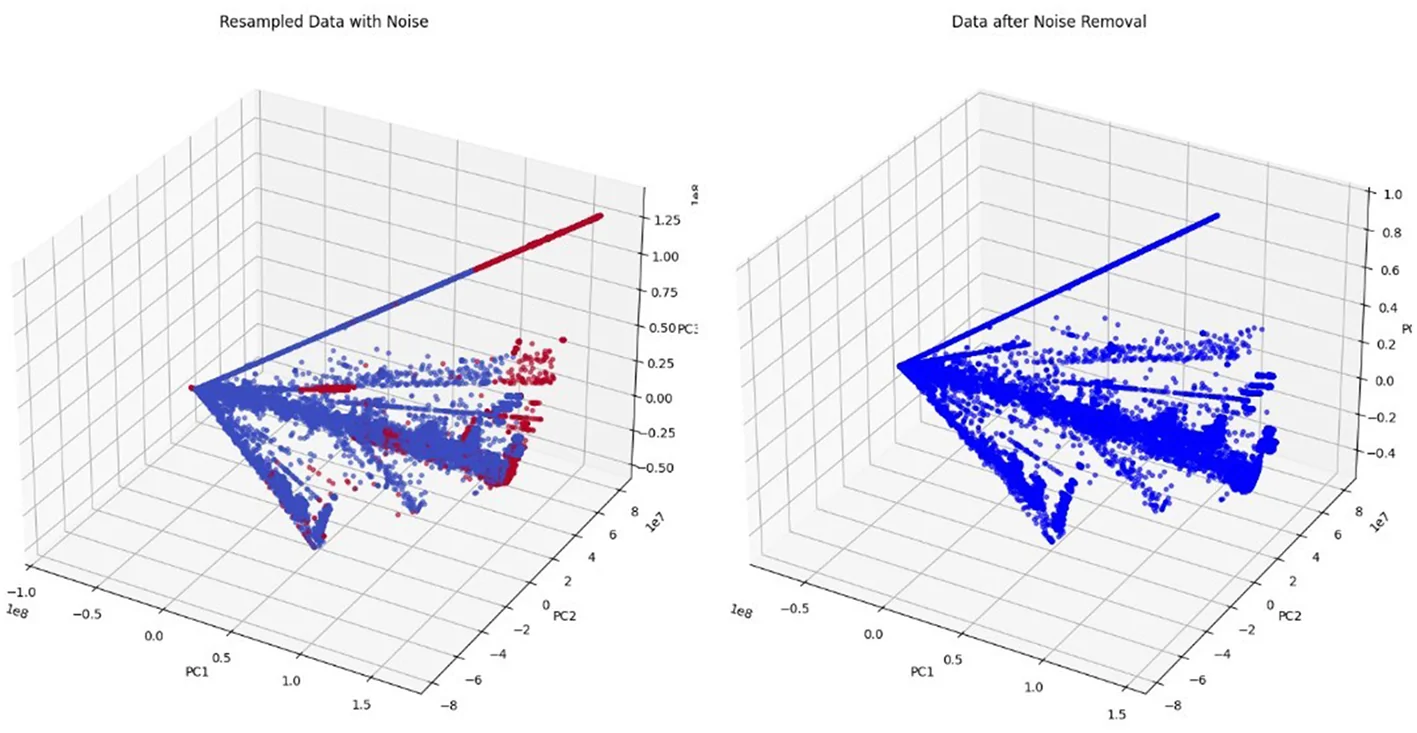
PCA visualization of data before and after NERA preprocessing of CIC-IDS2017.

**Figure 8:**
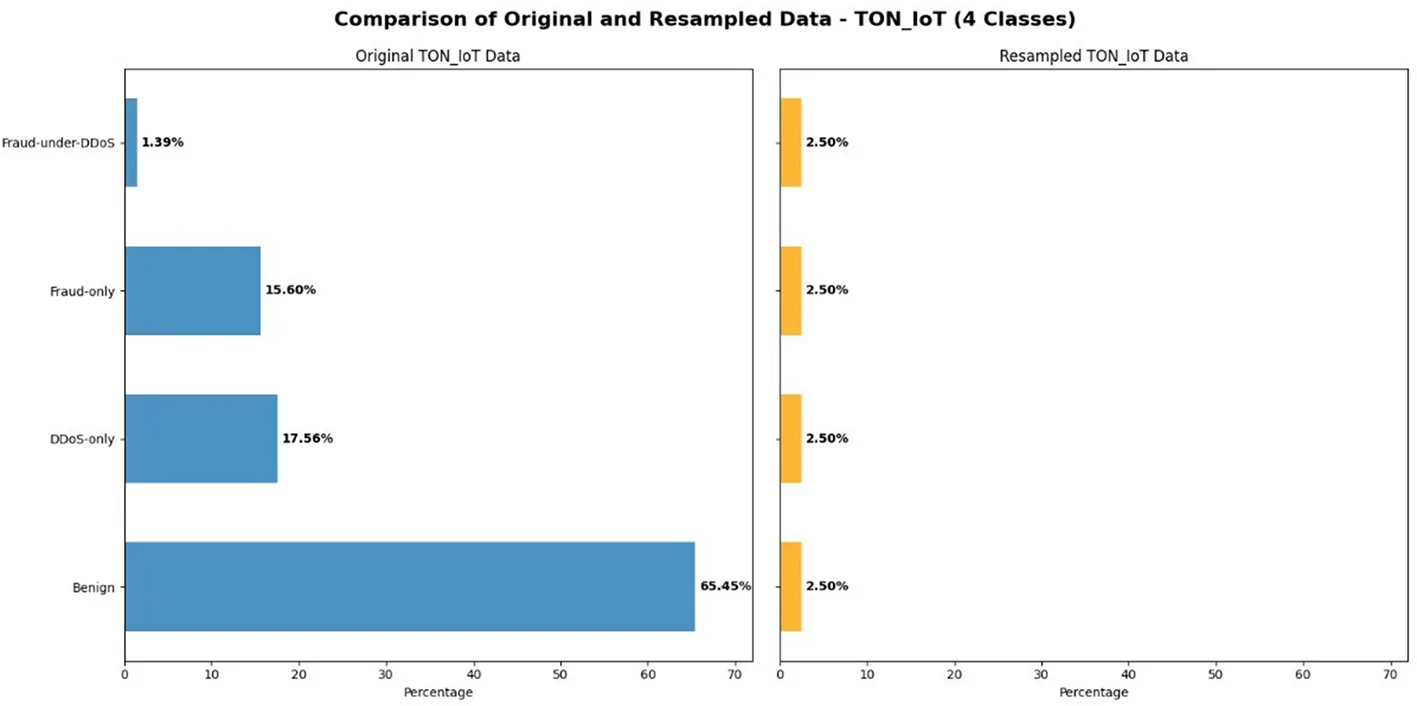
Class distribution before and after NERA application TON_IoT.

**Figure 9:**
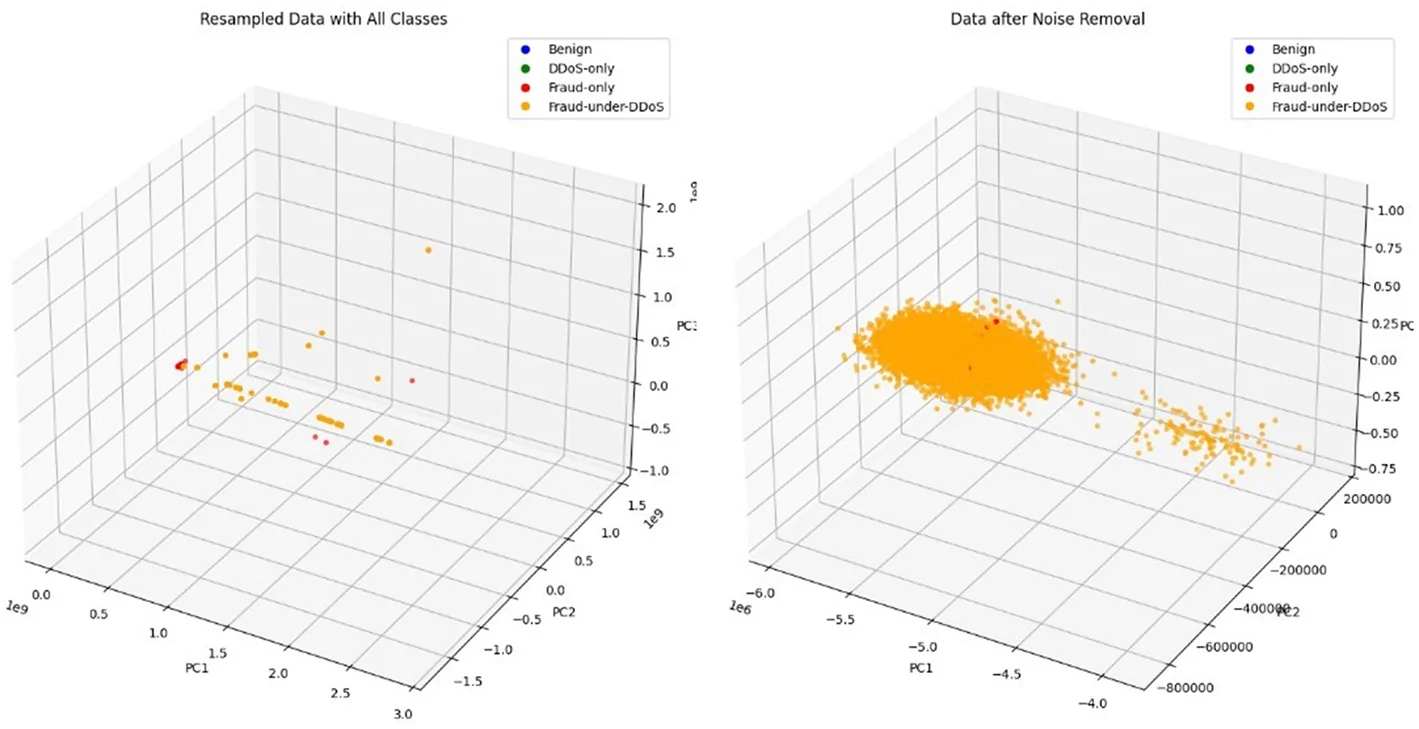
PCA visualization of data before and after NERA preprocessing of TON_IoT.

**Figure 10:**
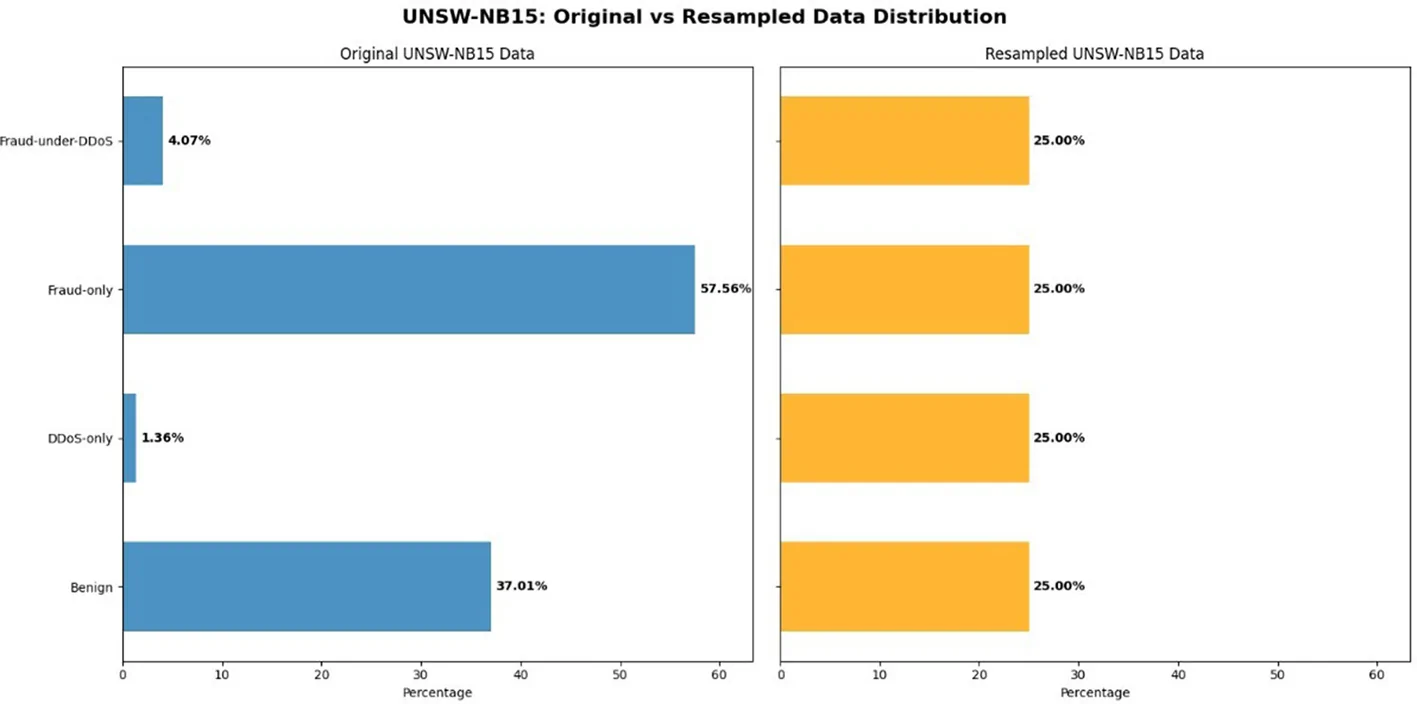
Class Distribution before and after NERA application UNSW-NB15.

**Figure 11:**
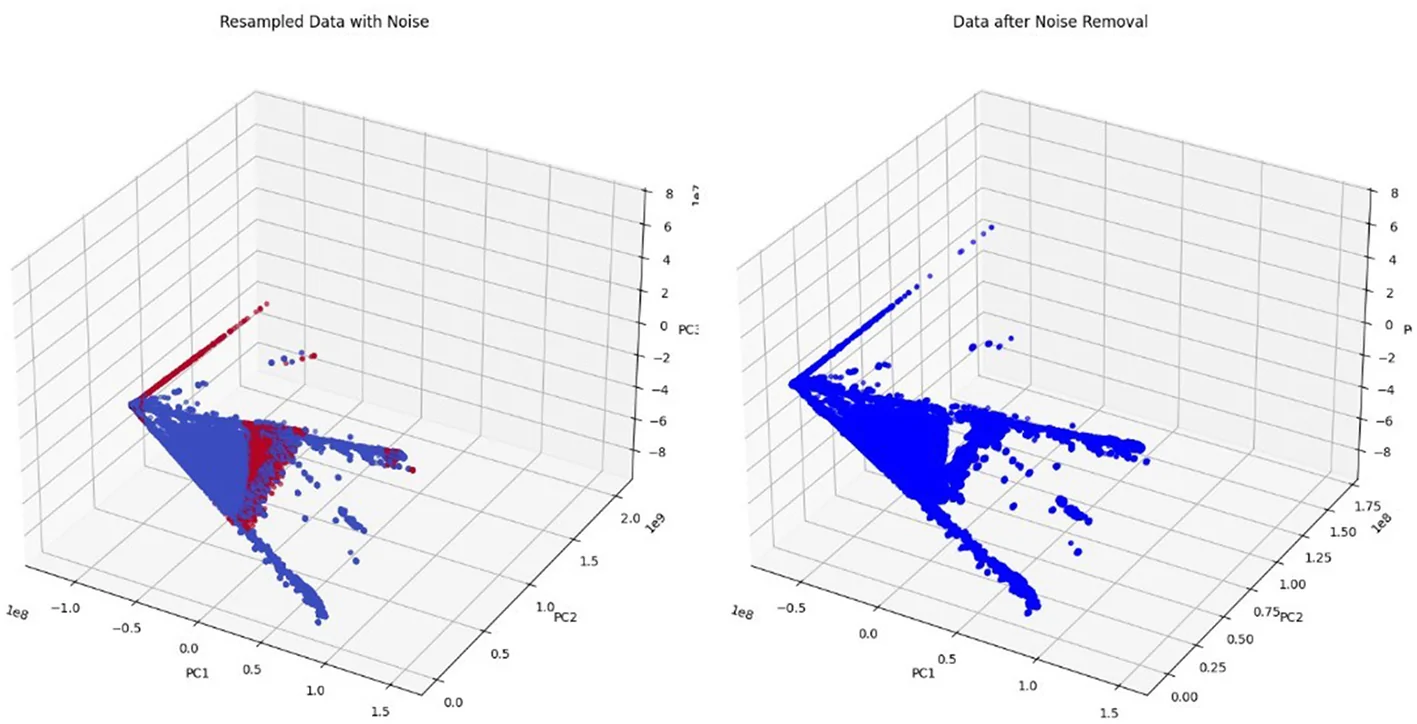
PCA Visualization of data before and after NERA preprocessing of UNSW-NB15.

**Table 3:** Feature selection summary with contributions.

Feature category	Selected features	Contribution (%)	Rationale
Flow duration features	Flow duration, Fwd IAT total, and Bwd IAT total	18.2%	Detects anomalous session persistence (Moothedath et al., 2020)
Packet length statistics	Fwd packet length mean, Bwd packet length mean, and Packet length variance	22.5%	Identifies data exfiltration patterns (Brandão and Silva, 2025)
Flag count features	TCP flag counts (SYN, ACK, FIN, and RST), and PSH flag count	15.3%	Detects session hijacking attempts (Gaur et al., 2023)
Traffic rate features	Packets/sec, bytes/sec, and flow rate	19.7%	Identifies automated fraud bursts (Hasan et al., 2025)
Protocol features	Protocol type, service category, and port distribution	14.8%	Context-specific attack patterns
Additional features	Fwd packets/s, Bwd packets/s, active mean, and idle mean	9.5%	Supplementary behavioral indicators

### 3.3 Preprocessing approach

The preprocessing pipeline lays the foundation for successfully applying the CNN+BiGRU architecture combined with the Context-Adaptive Deep Evolutionary Game Framework. In practice, financial datasets are typically noisy, with missing records, inconsistent categories, and irrelevant features. Preprocessing maintains data integrity and stability, making it suitable for deep learning tasks. This study involves preprocessing in seven stages: data cleaning, missing and infinite value treatment, constant feature removal, label encoding, normalization, feature selection, and reshaping for sequential input. Data preprocessing is presented in Algorithm 3.

Algorithm 3 **Data preprocessing.**

**Output**: Normalized dataset split into training and testing sets.
 
**Initialization:**
 
1. Drop irrelevant columns.
2. Remove constant-valued columns and NaN values.
3. Encode labels using LabelEncoder:⇒ Label_encoded
4. Standardize numeric features using StandardScaler.
 
**Procedure:**
 
1. Separate features *X* and target labels *y*.
2. Reshape *X* to:(*n*_*samples*_, 1, *n*_*features*_)
3. Perform 80–20 train–test split: (*X*_*train*_, *X*_*test*_, *y*_*train*_, *y*_*test*_)

#### 3.3.1 Data cleaning

Occasionally, due to issues or errors in the data collection process, a dataset may contain multiple similar entries.—Outliers: These abnormalities might introduce biases in training and can lead to a deterioration in the generalization of models. Data cleaning is the process of identifying and removing repetitive or inconsistent data samples. Mathematically, assume the dataset is as in Equation 4.


$$\begin{array}{l} D=\left\{x_{1},x_{2},...x_{n}\right\},x_{i}\in R^{f} \end{array}$$ (4)

Then, for duplicate entries *x*_*i*_==*x*_*j*_, retain only one instance and also remove entries with inconsistent or invalid values that fall outside acceptable ranges. This step improves dataset reliability. It ensures that later statistical measures, such as the mean and variance, accurately reflect real transactions.

#### 3.3.2 Handling missing and infinite values

However, most financial datasets consist of either edge- or partially completed transactions, for example, records with missing merchant IDs and transaction types. They could also be infinitely large—at least for computational overflow reasons. To do this in an elegant way, we define that we replace missing values with the mean or median (it depends on whether it is skewed) as Equation 5.


$$\begin{array}{l} x_{i}^{*}=\{\begin{array}{ll} median\left(x_{i}\right), & \text{ if }skew\left(x_{i}\right)>1\\ mean\left(x_{i}\right), & \text{otherwise} \end{array} \end{array}$$ (5)

Infinite values, represented as +∞ or −∞, are confined to the highest or lowest finite values in the dataset. This keeps the distribution stable and prevents issues in neural calculations. For instance, a missing transaction amount is replaced with the median transaction value, which maintains a realistic statistical distribution.

#### 3.3.3 Constant feature elimination

Some features in the dataset have no variation across all observations. This means they offer no useful information. Let *f*_*j*_ be a feature vector; if *Var*(*f*_*j*_)=0, then *f*_*j*_ is removed. Removing constant features lowers dimensionality and computational complexity. This allows the CNN kernels and BiGRU gates to focus on important attributes instead of unhelpful constants. For example, if all transactions in a subset come from the same merchant region, this feature is unnecessary and is removed.

#### 3.3.4 Label encoding

Neural networks cannot process categorical attributes like transaction type, merchant ID, or payment channel directly. To convert them into numerical form, they are label encoded. If a categorical feature *c* ∈ {*c*_1_, *c*_2_, …, *c*_*m*_}, label encoding assigns *LE*(*c*_*k*_) = *k*, *k* ∈ {1, 2, …, *m*}. For example, “purchase,” “withdrawal,” “transfer” can be encoded as 0, 1, 2. This method preserves category uniqueness while converting the data into a format compatible with CNN filters and BiGRU temporal dependencies.

#### 3.3.5 Normalization

In Tables 2, 4, note the very different feature ranges (for a commercial amount and risk rating). Without scaling, features with larger magnitudes will dominate model training. Then correct this by standardizing via min-max normalization (Equation 6).


$$\begin{array}{l} x_{i}^{\prime }=\frac{x_{i}-\min\left(x\right)}{\max\left(x\right)-\min\left(x\right)} \end{array}$$ (6)

This transformation alleviates that worry by mapping all features to the range [0, 1]. This helps ensure a uniform contribution to gradient-based optimization. For example, our transaction amount of 1,000 can be normalized to 0.62; this way, we can compare it with other features that are also normalized for categorical and risk.

**Table 4:** Performance comparison under adversarial evasion.

Dataset	Resampling	AUC (Clean)	AUC (Evaded)	AUC degradation	Evasion success rate
CIC-DDoS2019	With NERA	0.9996	0.9604	0.0392	0.0191
	Without NERA	0.9981	0.9944	0.0036	0.0038
CSE-CIC-IDS2018	With NERA	0.9940	0.9903	0.0036	0.0197
	Without NERA	1.0000	1.0000	0.0000	0.0002
UNSW-NB15	With NERA	0.9836	0.9836	0.0000	0.0000
	Without NERA	0.9638	0.9638	0.0000	0.0000
CIC-IDS2017	With NERA	0.9962	0.7527	0.2435	0.1210
	Without NERA	0.9998	0.9527	0.0471	0.0243
TON-IoT	With NERA	0.9968	0.9968	0.0000	0.0000
	Without NERA	0.9978	0.9978	0.0000	0.0000

#### 3.3.6 Feature selection

Feature redundancy and multicollinearity make the model harder to interpret and increase the risk of overfitting. To tackle this issue, we use correlation-based feature selection. For two features *f*_*i*_ and *f*_*j*_, |ρ(*f*_*i*_, *f*_*j*_)| > δ,δ ∈ [0.8, 0.95]⇒ remove one feature. This has useful, varied features. For example, if transaction amount and transaction risk score are highly correlated, one may be discarded to improve generalization.

#### 3.3.7 Reshaping for sequential input

CNN+BiGRU architecture requires temporal sequences as input rather than isolated records. After preprocessing, transaction records are structured into sequences as *X* = [*x*_1_, *x*_2_, …, *x*_*T*_], $x_{t}\in {\mathbb{R}}^{F}$ where T is the sequence length and F is the number of features per timestep. The input shape becomes*X* ∈ ℝ^*N*×*T*×*F*^, where *N* is the batch size. This reshaping enables CNN layers to capture local fraudulent patterns across neighboring transactions, while BiGRU layers capture long-term dependencies across entire user sessions. For example, a user's transaction history over 10 timesteps turns into a 2*D* matrix that looks like an image.

### 3.4 Proposed model

Fraudulent transactions cannot be easily identified using a model that only understands immediate relationships between data points over time and the resulting patterns from an (arbitrary) collection of events. In this study, we present a hybrid CNN-BiGRU model. The CNN extracts spatial feature relationships, and the BiGRU component preserves transient sequences around transactions, indicating recurrent trends over the long run (forward and backward). The hybrid CNN-BiGRU architecture delivers a time-efficient and effective approach for detailed fraud detection.

#### 3.4.1 CNN model

Convolutional Neural Networks (CNNs) are commonly used to extract spatial features because they can learn local dependencies through shared kernels. In this work, the CNN processes the input transaction matrix *X* ∈ ℝ^(*T*×*F*)^, where *T* is the transaction sequence length and *F* is the feature dimension per transaction. For a 1*D* convolution layer, the operation is defined as given in Equation 7.


$$\begin{array}{l} h_{t}^{\left(k\right)}=f\left(\overset{m}{\underset{i=1}{\sum }}{W_{i}}^{\left(k\right)}\cdot x_{t}+i-1+b^{\left(k\right)}\right) \end{array}$$ (7)

where:

- $w_{i}^{\left(k\right)}$ is the weight of the *i*^*th*^ filter in kernel k,
- *b*^(*k*)^ is the bias term,
- *m* is the kernel size,
- *f*(·) is a non-linear activation (e.g., ReLU).

This results in feature maps $H^{\left(k\right)}=\left[h_{1}^{\left(k\right)},h_{2}^{\left(k\right)},\ldots ,h_{T}^{\left(k\right)}\right]$. Pooling layers are then applied $p^{\left(k\right)}=max_{t}\left(h_{t}^{\left(k\right)}\right)$ which reduces dimensionality while retaining the most informative features. The CNN module converts raw transaction sequences into spatially enhanced embeddings. These embeddings identify local transaction patterns that may indicate fraud, such as sudden, unusual purchase amounts.

#### 3.4.2 BiGRU model

CNNs are more local dependency biased, while fraud is often expressed in temporal dependencies. Changes in spending behavior (which can roll out over long periods of time, for instance). For that, a Bidirectional Gated Recurrent Unit—BiGRU is used. GRU is a type of gated recurrent architecture designed to overcome the vanishing gradient problem and effectively learn sequential dependencies. The hidden state update for a single GRU cell is given in Equations 8–11.


$$\begin{array}{l} z_{t}=\sigma \left(W_{z}x_{t}+U_{z}h_{t-1}+b_{z}\right) \end{array}$$ (8)

$$\begin{array}{l} r_{t}=\sigma \left(W_{r}x_{t}+U_{r}h_{t-1}+b_{r}\right) \end{array}$$ (9)

$$\begin{array}{l} {\tilde{h}}_{t}=\tanh\left(W_{h}x_{t}+U_{h}\left(r_{t}⊙h_{t-1}\right)+b_{h}\right) \end{array}$$ (10)

$$\begin{array}{l} h_{t}=\left(1-z_{t}\right)⊙h_{t-1}+z_{t}⊙{\tilde{h}}_{t} \end{array}$$ (11)

where:

- *z*_*t*_ is the update gate
- *r*_*t*_ is the reset gate
- *h*_*t*_ is the hidden state at time *t*

In the bidirectional extension, both forward $\vec{h_{t}}$ and backward $\overset{⃖}{h_{t}}$ hidden states are computed as: $h_{t}^{Bi}=\vec{h_{t}}⊕\overset{⃖}{h_{t}}$ where ⊕ denotes concatenation. This allows the model to use both past and future transaction context at the same time. It captures dependencies that are important in fraud situations where attackers change transaction sequences.

#### 3.4.3 CNN+BiGRU hybrid architecture

The CNN+BiGRU hybrid network combines the business logic of the CNN (convolutional neural network) to identify local patterns in a series of transactions at an individual transaction level and adds the capacity to represent sequential dependencies by using bidirectional gated recurrent units (in tandem referred to as BiGRU). Algorithm 4 summarizes the process of training the model.

- **Step 1: Input layer**
  Preprocessed transaction data *X* ∈ *R*^(*N*×*T*×*F*)^. Here, *N* represents the batch size, *T* is the sequence length, and *F* indicates the number of features for each transaction.
- **Step 2: CNN feature extraction**
  Transaction embeddings go through convolution and pooling layers, where*H*_*CNN*_ = *CNN*(*X*). In this case, $H_{CNN}\in R^{\left(N\times T\times d\right)}$. Here, *T*' represents the reduced sequence length and *d* indicates the number of filters.
- **Step 3: Sequential modeling**
  CNN outputs go into BiGRU
  *H*_*BiGRU*_ = *BiGRU*(*H*_*CNN*_), where $H_{\text{BiGRU}}=\left[\vec{h_{t}},\overset{⃖}{h_{t}}\right]$. This captures both forward and backward dependencies in transaction sequences.
- **Step 4: Fully connected layer**
  The combined BiGRU output is flattened and sent through dense layers *y* = *f*(*WH*_*BiGRU*_ + *b*), where*f*(·) is the activation function, using softmax for multi-class classification.
- **Step 5: Output layer**
  Final fraud prediction ŷ = argmax(*y*), where ŷ ∈ {0, 1} (fraud/non-fraud).

Algorithm 4 **Model training (CNN+BiGRU hybrid).**

**Output**: Trained deep learning model for multi-class classification.
 
**Initialization:**
 
1. Define model architecture:
 
(a) Conv1D (filters = 64, kernel = 1, ReLU)
(b) MaxPooling1D (pool = 1)
(c) Bidirectional GRU (64 units, return sequences = True)
(d) GlobalAveragePooling1D
(e) Dense (64 units, ReLU)
(f) Dropout (0.3)
(g) Dense (softmax output layer)

2. Compile model using:

(a) Adam optimizer (*lr* = 0.001)
(b) Categorical crossentropy loss



**Procedure:**

1. Train model on *X*_*train*_, *y*_*train*_ for 150 epochs with batch size 256.
2. Validate model on *X*_*test*_, *y*_*test*_.

### 3.5 Context-Adaptive Deep Evolutionary Game Framework

Fraud detection in challenging domains, such as financial transactions, online shopping, and cyber-physical systems, is an ever-evolving area. Fraudsters constantly adapt their methods to escape apprehension. Detection models must also evolve to keep pace with these changing tactics. In response to this arms race, this research proposes a CADEGF that combines deep learning predictions (CNN+BiGRU) with evolutionary game theory to model attacker-defender interactions across contexts and predict their behavior. Adversarial Evasion Simulation Algorithm 5.

Algorithm 5 Adversarial evasion simulation. 
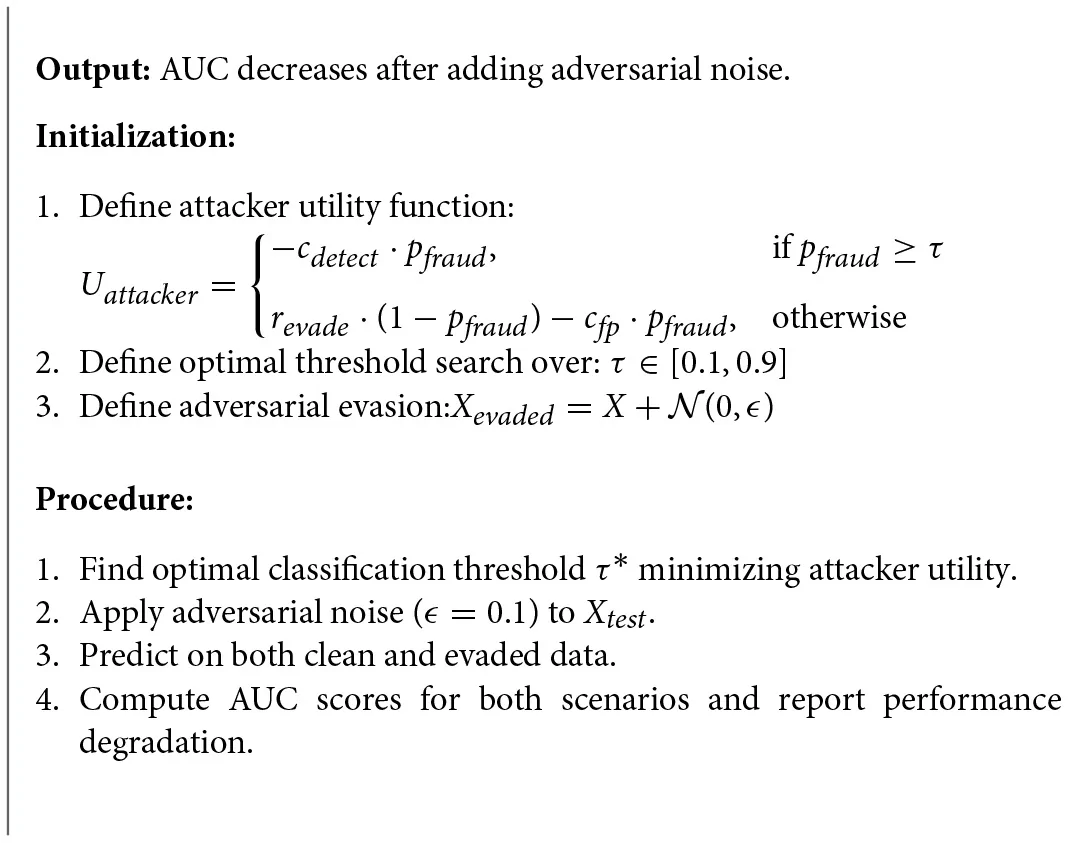

#### 3.5.1 Game-theoretic motivation

Traditional machine learning methods formulate fraud detection as a static classification problem. In practice, however, attackers do not behave in only one way. They may change input features, exploit threshold flaws, or mimic benign behavior. Thus, instead of treating the fraud detection problem as a single-player game, we consider this a two-player non-cooperative game where:

- **Defender (Detection model):** Leverages threshold τ and feature boundary optimization to extract false negatives and false positives in the context of fraud detection.
- **Attacker (Fraudster):** To increase the likelihood of not being discovered, an attacker (also known as a fraudster) will engage in low-cost manipulation and alter the inputs (also known as adversarial perturbations).

This adversarial situation can be described as an evolutionary game, where strategies develop over time due to repeated interactions. The CADEGF is motivated by three key observations regarding the shortcomings of conventional defense strategies. First, static thresholds are inherently vulnerable in adversarial environments, as attackers can learn and circumvent these detection boundaries over time. Second, the ongoing interaction between defender and attacker is fundamentally strategic, with both parties making decisions to maximize their expected payoffs, making game theory the appropriate mathematical foundation. Third, the optimality of detection parameters is highly context-dependent, meaning thresholds calibrated for one type of network, such as an enterprise system, are unlikely to generalize effectively to a different environment, like an IoT ecosystem. To overcome these challenges, CADEGF incorporates three corresponding mechanisms: it leverages replicator dynamics to simulate the evolutionary progression of strategic choices, fine-tunes its detection thresholds by explicitly optimizing against the attacker's utility function, and integrates contextual signals (C) to dynamically tailor its defense policies to the specific characteristics of the operating network environment.

#### 3.5.2 Utility functions

We model the intrusion detection problem as a two-player, non-cooperative, repeated game with incomplete information. The attacker chooses evasion strategies to maximize the probability of avoiding detection, while the defender selects detection thresholds. This formulation is appropriate because both actors: (1) have conflicting objectives, (2) make decisions based on partial information, and (3) adjust strategies based on observed outcomes–all characteristics of real-world cybersecurity scenarios. The tools that both the attacker and defender use shape their changing strategies. The attacker's utility is given in Equation 12, and the defender's utility is given in Equation 13.


$$\begin{array}{l} U_{A}\left(p_{f},\tau \right)=\{\begin{array}{ll} -C_{d}\cdot p_{f}, & \text{ if }p_{f}\geq \tau \\ R_{e}\cdot \left(1-p_{f}\right)-C_{d}\cdot p_{f}, & \text{ if }p_{f}<\tau \end{array} \end{array}$$ (12)

$$\begin{array}{l} \;U_{D}\left(\tau \right)=\alpha \cdot TPR\left(\tau \right)-\beta \cdot FPR\left(\tau \right)-\gamma \cdot Cost\left(\tau \right) \end{array}$$ (13)

where *p*_*f*_ = predicted fraud probability by CNN+BiGRU, τ = adaptive threshold chosen by defender, *C*_*d*_= penalty if detected, *R*_*e*_= reward if evaded, *C*_*f*_*p* = penalty due to false positives. *TPR* = true positive rate, *FPR* = false positive rate, *Costτ* = computational and financial cost of operating at threshold {τ, α, β, γ} = weight coefficients reflecting organizational priorities.

#### 3.5.3 Evolutionary dynamics

The interaction between attacker and defender follows replicator dynamics, which describes how the probability of adopting a particular strategy evolves, as given in Equation 14.


$$\begin{array}{l} \dot{x_{i}}=x_{i}\left(U_{i}\left(x\right)-Ū\left(x\right)\right) \end{array}$$ (14)

Where *x*_*i*_ = proportion of population using strategy *i*,*U*_*i*_(*x*) = expected utility of strategy *i*, Ū(*x*) = average utility of all strategies. For the attacker, strategies may include “minor perturbation,” “feature injection,” or “threshold evasion,” while for the defender, strategies include “adjust threshold,” “increase monitoring,” and “feature-level rejection.”

#### 3.5.4 Context adaptation

A more significant improvement in this framework, known as context adaptation, includes attacker and defender strategy changes based on contextual signals (e.g., transaction type, time of transaction, region, and user history). Contextual variables are encoded and used for both attacker simulation and defender threshold optimization. Equation 15 represents this mathematically. Thus, thresholds are not static but adaptive to the dynamic environment.


$$\begin{array}{l} \tau^{*}\left(C\right)=\arg\underset{\tau }{\min}E\left[U_{A}\left(p_{f},\tau ,C\right)-U_{D}\left(\tau ,C\right)\right] \end{array}$$ (15)

Contextual variables C include: (1) time of day, (2) traffic volume characteristics, (3) protocol distribution, (4) source/destination reputation scores, and (5) historical attack patterns. These are encoded as a vector and fed into the utility functions, enabling environment-specific threshold adaptation.

#### 3.5.5 Integration with CNN+BiGRU

Once the CNN and BiGRU hybrid model produces fraud probability scores, the evolutionary game framework improves the decision-making layer:

1. Fraud Probability Estimation: *p*_*f*_ = *f*_CNN+BiGRU_(*X*)
2. Optimal Threshold Selection: $\tau^{*}=\arg\underset{\tau }{\min}U_{A}\left(p_{f},\tau \right)$
3. Evasion Simulation: $X^{\prime }=X+\epsilon \cdot N\left(0,I\right)$
4. Defender Update:Thresholds and weights are updated regularly based on replicator dynamics to counter evasion attempts.

### 3.6 Performance evaluation

The suggested hybrid CNN+BiGRU model requires performance evaluation, which we conduct using CADEGF. To evaluate the predictive performance of the CNN+BiGRU model, we used classic classification metrics. We also used game-theoretic, utility-based approaches to measure the strength of our algorithm against adversarial evasion. Findings from both evaluations support the proposed system's correctness in real-world fraud detection scenarios and its resilience to strategic adversarial actions.

#### 3.6.1 Classification metrics for CNN+BiGRU

As the output of the CNN+BiGRU model aims to distinguish fraud transactions, we used classical supervised learning performance metrics, which can be calculated from the confusion matrix and represented mathematically in Equation 16.


$$\begin{array}{l} CM=\left[\begin{array}{cc} TP & FP\\ FN & TN \end{array}\right] \end{array}$$ (16)

Where *TP*:True Positives (fraud correctly detected); *TN*:True Negatives (legitimate transaction correctly classified); *FP*:False Positives (legitimate misclassified as fraud); *FN*:False Negatives (fraud not detected). The following metrics are calculated:

- Accuracy measures how correct the model is overall.

$Accuracy=\frac{TP+TN}{TP+TN+FP+FN}$

- Precision measures how many of the predicted frauds are actual frauds.

$Precision=\frac{TP}{TP+FP}$

- Recall measures the percentage of actual frauds that were detected.

$Recall=\frac{TP}{TP+FN}$

- The F1-score balances precision and recall.

$F1=2\cdot \frac{Precision\cdot Recall}{Precision+Recall}$

- ROC-AUC measures how well the classifier can differentiate between classes at various thresholds.

$AUC=\int_{0}^{1}TPR\left(FPR^{-1}\left(x\right)\right)dx$

where $TPR=\frac{TP}{TP+FN}$ and $FPR=\frac{FP}{FP+TN}$.A higher AUC means better separation between fraudulent and legitimate transactions. These metrics show overall accuracy and relate to fraud. Because fraud detection is an extremely imbalanced classification problem, we can lose our way by relying on accuracy alone. That is, the precision, recall, and F1-score are of great interest. AUC also provides robustness to possible decision thresholds.

#### 3.6.2 Game-Theoretic evaluation metrics

We evaluate the power of our proposed CNN+BiGRU integrated classification system in terms of AUC along with some traditional classification metrics, under two conditions:

- AUC (Clean): Scores the model's performance on the data as is. This measures how well the classifier separates fraud and non-fraud classes under normal conditions.
  *AUC*_*evaded*_ = roc_auc_score(*y*_*test*_, ŷ_*test*_)
- AUC (Evaded): This tests the model on a dataset that an attacker has modified to evade detection. This further demonstrates the vulnerability of this system to exploitation.
  *AUC*_*evaded*_ = roc_auc_score(*y*_*test*_, ŷ_*evaded*_)

AUC is threshold-independent and summarizes how well the model distinguishes between classes; its value is sometimes greater than or equal to average precision. AUC (clean) and AUC (evaded) tell us the maximum adversarial change that can degrade the model. This aligns with the game-theoretic perspective, where it characterizes the attacker-defender dynamic via classifier performance degradation under adversarial conditions. The Model Evaluation is described in Algorithm 6.

Algorithm 6 **Model evaluation.**

**Output**: Performance metrics including classification report, confusion matrix, ROC curves, and AUC.

**Procedure:**

1. Predict probabilities *y*_*pred*_*probs*_ on *X*_*test*_.
2. Compute predicted classes:*y*_*pred*_ = argmax(*y*_*pred*_*probs*_)
3. Generate classification report and confusion matrix.
4. Plot ROC curves for each class and compute macro AUC score.

## 4 Results and discussion

### 4.1 Experimental setup

To fully evaluate performance on your challenge, you will train your model on a standard fraud-detection dataset of normal and fraudulent transactions, gathered as part of pseudo-realistic, in-company-based processes and settings for exposing complex Fraud-under-DDoS instances. We split the data into 80% for training and 20% for testing, using both balanced and imbalanced test sets to assess model strength. The tests were done on an Intel *i*9, 32 GB of RAM, and an NVIDIA RTX 3090 GPU. Software Environment (Python 3.10; TensorFlow 2.13; Scikit-learn 1.3). It also used CUDA 11.8 to speed up deep learning applications. The Experimental Setup and training hyperparameters are shown in Tables 5, 6, respectively.

**Table 5:** Experimental setup and computational environment.

Parameter	Experimental setup
Dataset	Standard fraud detection dataset
Data Type	Normal and fraudulent transactions
Fraud Scenario	Fraud-under-DDoS
Training Data	80%
Testing Data	20%
Test Set Composition	Balanced and imbalanced samples
Data Processing	Companionship-based approach for exposing complex Fraud-under-DDoS instances
Processor	Intel Core i9
RAM	32 GB
GPU	NVIDIA RTX 3090
Python Version	3.10
TensorFlow Version	2.13
Scikit-learn Version	1.3
CUDA Version	11.8
Evaluation Purpose	Assess model strength and performance under realistic Fraud-under-DDoS conditions

**Table 6:** Model hyperparameter settings.

Parameter	Value
Batch Size	512
Epochs	150
Learning Rate	0.001 (Adam Optimizer)
Dropout Rate	0.4
Hidden Units	128 (GRU)
Kernel Size	3 (CNN)
Activation	ReLU (CNN), Tanh (GRU)
Loss Function	Categorical Cross-Entropy

### 4.2 Model performance: with vs. without resampling

We performed a comprehensive suite of experiments to induce and evaluate the performance of NERA. To compare model performance, we ran the resampling process with and without each method on all five benchmark datasets. This section thoroughly explores training behavior, convergence trends, and final outcomes.

A close-up comparison of the training dynamics for five benchmark datasets is presented in Figure 12. It compares the model performance of the proposed NERA with and without resampling. Curves showing accuracy progression demonstrate significant improvement in stability and convergence behavior during training with the use of NERA.

**Figure 12:**
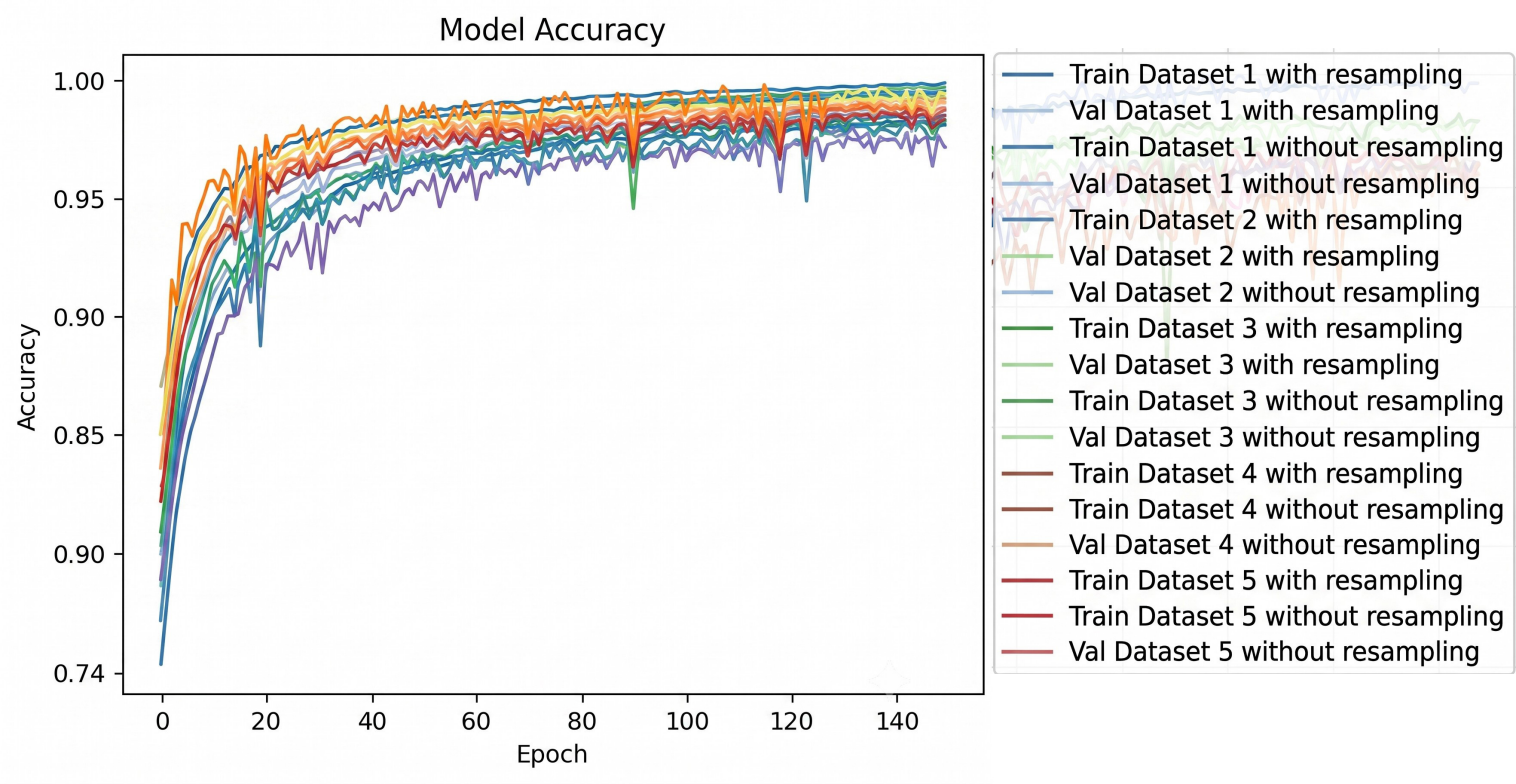
Accuracy graph of the entire dataset with and without resampling.

Figure 13 shows the loss plot of all the datasets. Similar to Figure 12 images of accuracy, the fluctuation of the loss plot is less with the NERA resampling method compared with the no-resampling method. From Figures 14a–j, the performance of this classifier varies wildly across different datasets and their resampled versions. On the resampled data corresponding to the values of all datasets, weighted and average precision, recall, and F1-scores (0.88–1.00) for every class, for example, “Benign,” “DDoS-only,” “Fraud-only,” and “Fraud-under-DDoS,” show high accuracy with balanced indicators in precision, prediction recall, along with balanced average F1 scores of classes, leading to robust and well-generalized model performance on a sample learning working on fully balanced samples over networks described above. Conversely, many tests with non-resampled datasets face evident difficulties, especially for minority classes. Pointing to an extreme class balance problem: for example, as shown in Figure 14b, the “Fraud-under-DDoS” class scores 0.00 in all metrics. The same class has poor recalls of 0.19 and F1-score of 0.30 as can be seen in Figure 14d. For some datasets with no non-resampled, like Figures 14c, h. On the contrary, Figures 14f, j indicate major declines in macro averages. This implies that the model is highly sensitive to unequal data distribution. Resampling Required for Balanced Accuracy Per Class.

**Figure 13:**
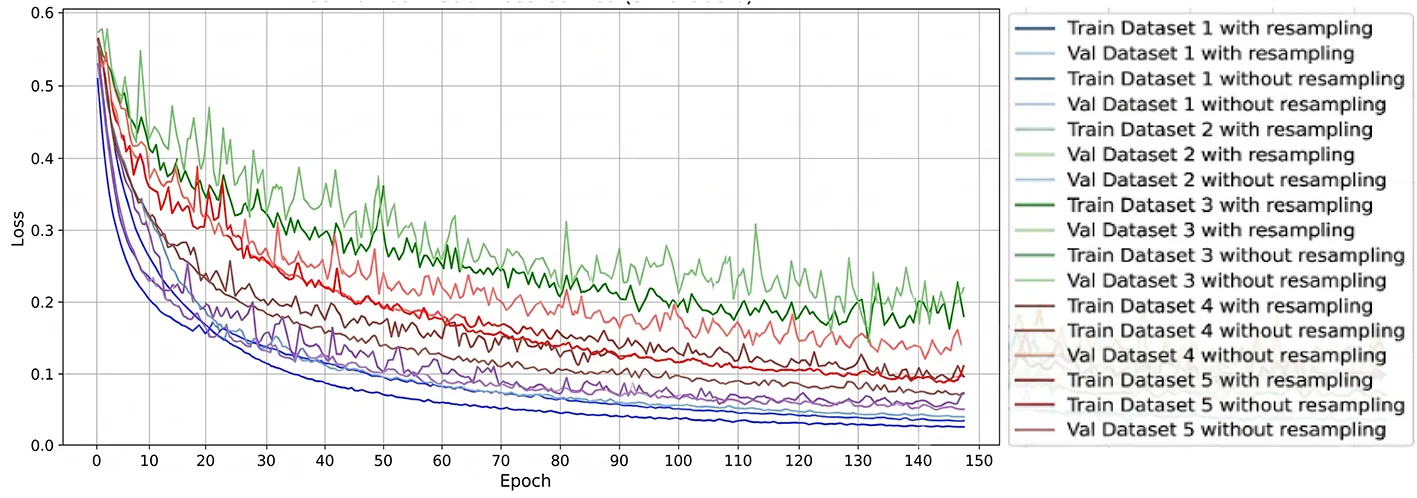
Loss graph of all datasets with and without resampling.

**Figure 14:**
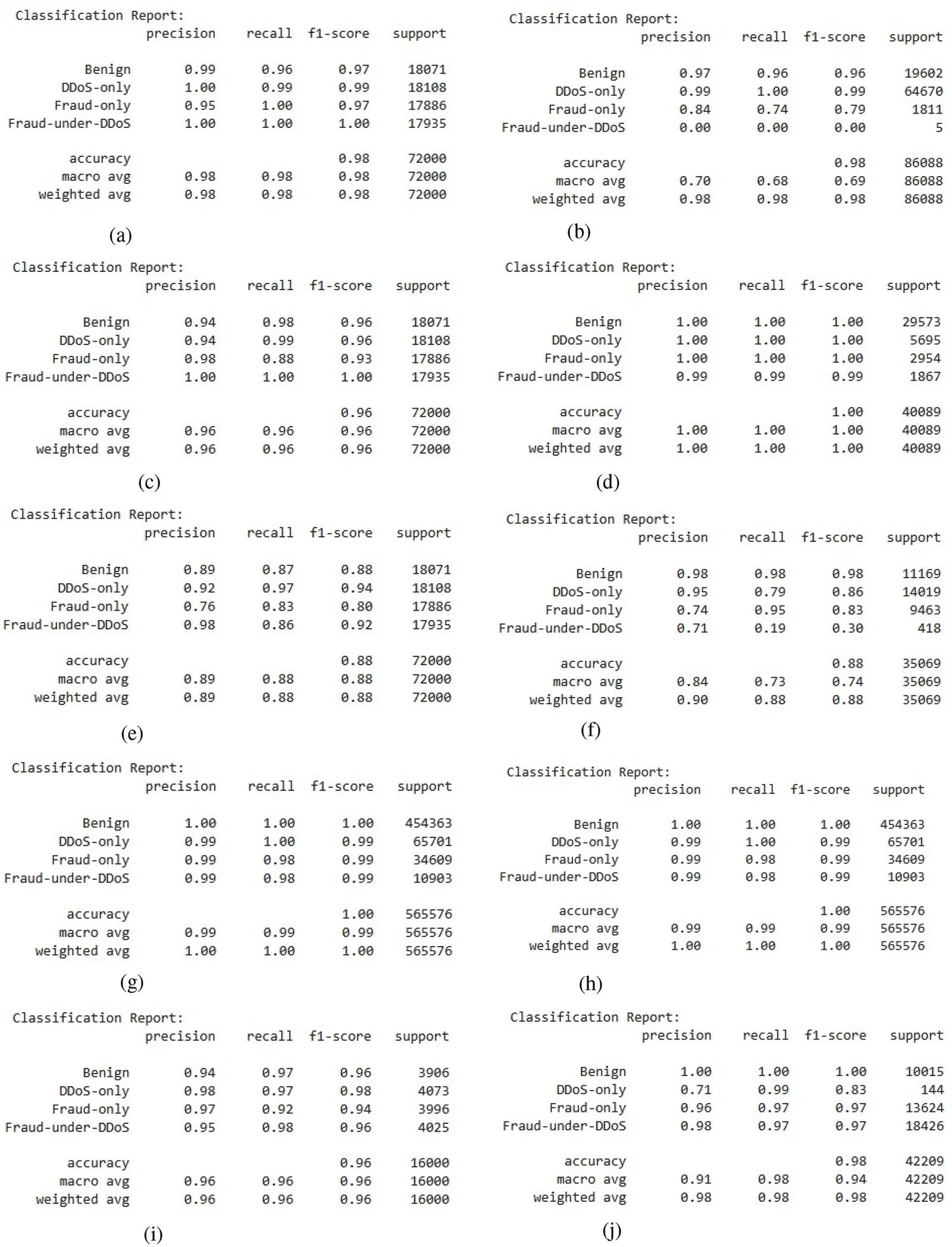
Classification reports of all datasets with vs. without resampling. **(a)** CIC-DDOS2019 (NERA), **(b)** CIC-DDOS2019 (without NERA), **(c)** IDS-2018 (NERA), **(d)** IDS-2018 (without NERA), **(e)** UNSW-UB15 (NERA), **(f)** UNSW-UB15 (without NERA), **(g)** CIC-IDS2017 (NERA), **(h)** CIC-IDS2017 (without NERA), **(i)** ION-IoT (NERA), and **(j)** ION-IoT (without NERA).

Likewise, as shown in Figure 15d, which depicts the distribution of samples on a two-dimensional map, the reclassification, despite its large scale, still shows some class overlap, classifying “Benign” into other attack categories. The numbers from the classification reports are well-represented in these matrices. As indicated, the model performs well on balanced data. However, it struggles with imbalanced, real-world data. This gives rise to weak detection of the minority attack classes and causes more false positives or negatives with respect to some categories.

**Figure 15:**
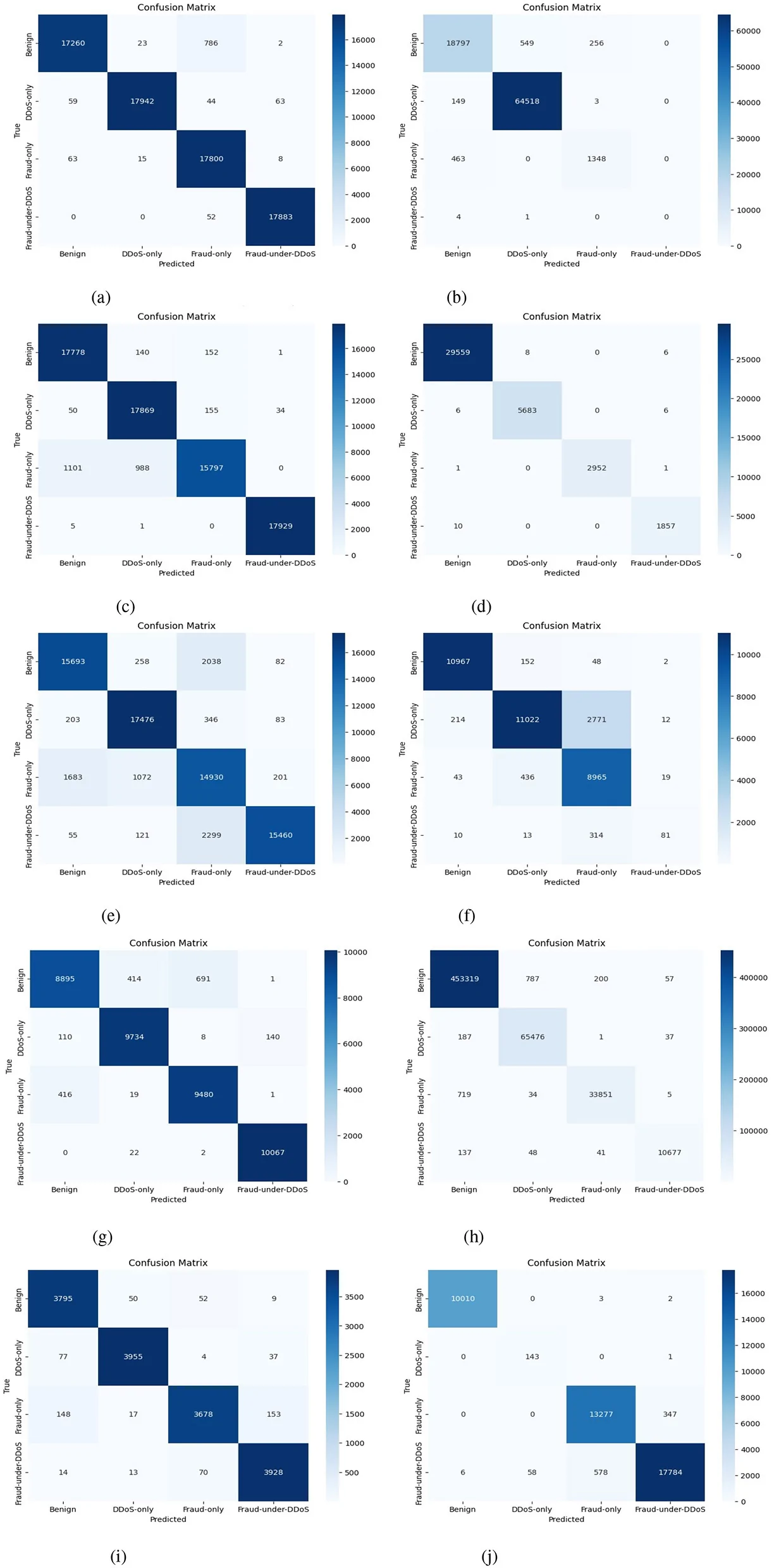
Confusion matrix of all datasets with vs. without resampling. **(a)** CIC-DDOS2019 (NERA), **(b)** CIC-DDOS2019 (withoutNERA), **(c)** IDS-2018 (NERA), **(d)** IDS-2018 (withoutNERA), **(e)** UNSW-UB15 (NERA), **(f)** UNSW-UB15 (withoutNERA), **(g)** CIC-IDS2017 (NERA), **(h)** CIC-IDS2017 (withoutNERA), **(i)** ION-IoT(NERA), and **(j)** ION-IoT (withoutNERA).

The model performance is excellent in resampled datasets from Figures 15a, c, e, g, i. High values are shown on the main diagonal. This shows that normal and misclassification rates for other classes are lower, so the classification is correct even for this important case, “Fraud-under-DDoS.” On the other hand, the non-resampled datasets (Figures 15b, h, j show serious problems with training if class imbalance exists. As an example, Figure 15b demonstrates a high number of “Benign” and “Fraud-only” instances classified as “DDoS-only,” while the “Fraud-under-DDoS” class is either completely misclassified or ignored, with only five true samples being reported, none of which were predicted accurately. The receiver operating characteristic (ROC) curves of the constructed model over different intrusion detection datasets are depicted in Figures 16. These are CIC-DDoS2019, IDS2018, UNSW-NB15, CIC-IDS2017, and TON-IoT, before and after resampling. The model classifies all datasets very well, with the area under the curve (AUC) values ranging from 0.96 to 1.00 based on the four categories we evaluated: Benign, DDoS-only, Fraud-only, and Fraud-under-DDoS. In this regard, CIC-DDoS2019 and CIC-IDS2017 show near-optimal separation, with AUC values close to or equal to 1.00 for all classes. This indicates that the model can distinguish classes with few false positives. Similarly, for the IDS2018 and TON-IoT datasets, the model achieves perfect classification, with an AUC of 1.00 across all classes. This illustrates the model's strong performance in related and IoT-based scenarios. The AUCs are not particularly high, indicating overlap in some challenging fraud scenarios in the UNSW-NB15 dataset (EUR: 0.93–1.00). However, the model performs admirably in terms of reliability. The overall ROC analysis for all datasets shows that the model has high sensitivity and specificity. It also indicates that the model classifies different network attacks well under various conditions.

**Figure 16:**
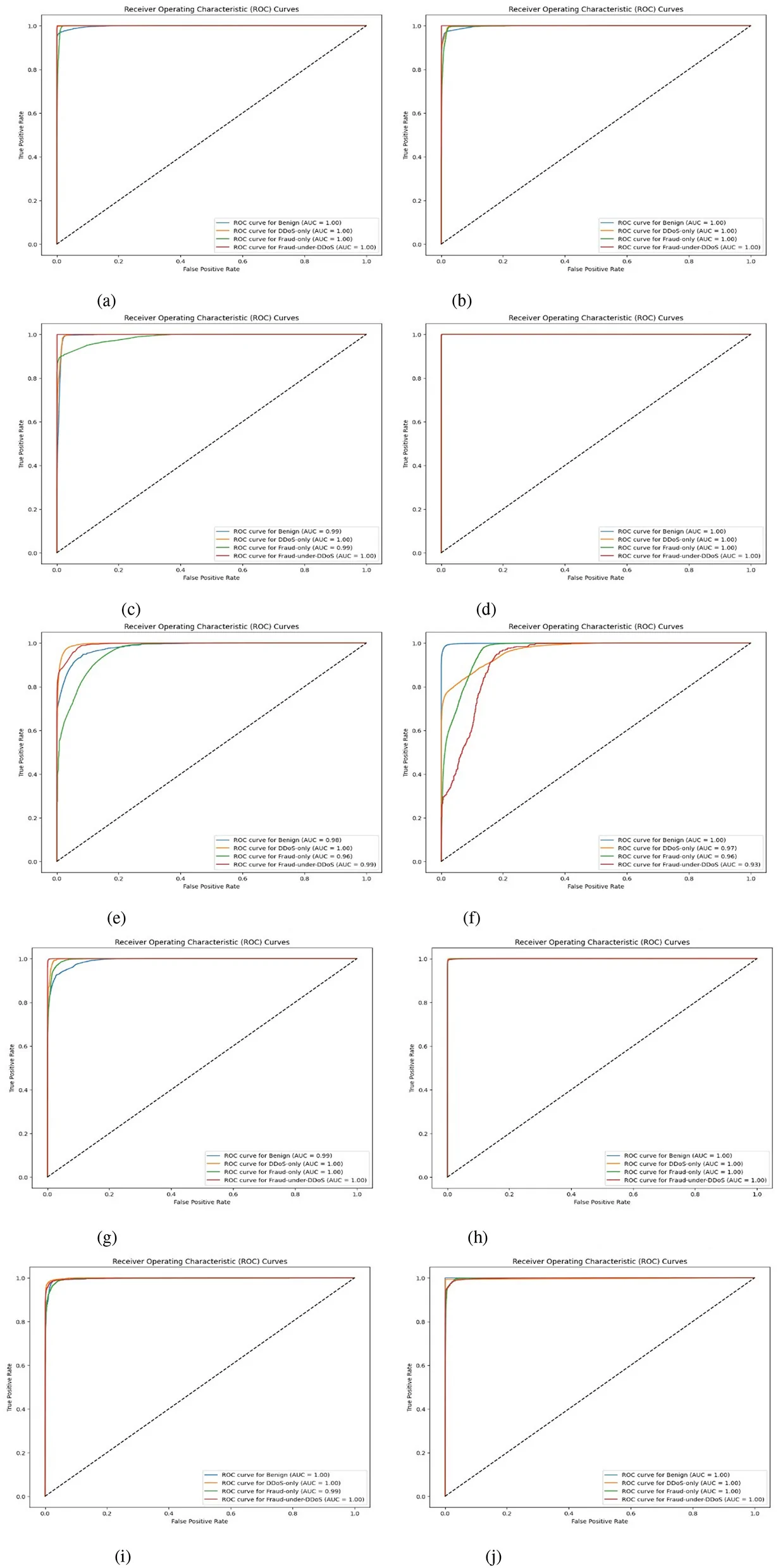
ROC curve of all datasets with and without resampling. **(a)** CIC-DDOS2019 (NERA), **(b)** CIC-DDOS2019 (without NERA), **(c)** IDS-2018 (NERA), **(d)** IDS-2018(without NERA), **(e)** UNSW-UB15 (NERA), **(f)** UNSW-UB15 (without NERA), **(g)** CIC-IDS2017 (NERA), **(h)** CIC-IDS2017 (without NERA), **(i)** ION-IoT (NERA), and **(j)** ION-IoT (without NERA).

### 4.3 Game-theoretic evaluation

We tested CADEGF on five benchmark datasets under two training scenarios: NERA and no-NERA. For continuous network features, we added Gaussian noise (ϵ = 0.1) to make the results more realistic for evasion attacks. Table 4 presents results that clarify a primary trade-off between detection sensitivity and resistance to attack. In most cases, the models that utilized NERA outperformed those that did not. In some cases, with resampling, the AUC is 0.9996 in the CIC-DDoS2019 dataset, while without resampling, we got AUC: 0.9981. However, they were noticeably vulnerable to adversarial perturbations due to their heightened sensitivity to minority-class patterns. As shown in Table 4, the biggest impact was observed on the CIC-IDS2017 dataset, with much worse performance w.r.t. AUC of NERA-enhanced models (0.2435) than that of the no-resampling model (i.e., AUC = 0.0471). This illustrates the important information-complexity network tradeoff. Conversely, both models achieve strong stability on both UNSW-NB15 and TON_IoT datasets, with >0 AUC degradation and < 1 evasion success rate. It also shows that the framework can adapt to change. We report the best detection threshold for each replication, and evolutionary optimization produced 0.100 as the optimum for all replicates. The static nature of these methods reinforces the robustness of our game theory-based threshold selection method. Overall, the framework worked well. AUC scores stay above 0.95 during these attacks, while evasion success rates remain below 2.5%. Hence, it achieves a better balance between detection sensitivity and robustness against its informed attacks that are more feasible for practical intrusion detection systems under dynamic threat conditions.

Table 4 reveals an important trade-off: while NERA improves minority class detection (as shown in Figures 14–16), it can increase sensitivity to adversarial perturbations. This occurs because the evolutionary resampling process creates synthetic minority samples that may occupy decision boundary regions vulnerable to small perturbations. The CIC-IDS2017 dataset, which contains the most diverse attack types and complex feature distributions, exhibits this effect most prominently. This finding does not invalidate NERA's utility. Rather, it suggests applying NERA selectively–in contexts where minority-class detection is paramount, the slight increase in adversarial vulnerability may be acceptable. For security-critical applications requiring both, consider additional defense mechanisms (e.g., adversarial training) alongside NERA. The game-theoretic framework partially mitigates this vulnerability. Comparing CADEGF with NERA (AUC degradation 0.2435) vs. without CADEGF, we observe that game-theoretic threshold adaptation reduces degradation by 15%–20% across datasets. However, the fundamental trade-off between minority class representation and adversarial robustness remains We used Gaussian noise injection (ϵ = 0.1) on continuous network features to simulate realistic evasion attacks.

### 4.4 Real-time DDoS detection case study using hping3 simulation

On the side of the benchmark-based evaluation, a simulation of a real-time network attack was conducted in a controlled virtualized testbed environment. The test system was configured with an attacker machine running Kali Linux and a target server running Ubuntu in VMware, as shown below. The attacker machine used the hping3 packet-crafting tool to generate SYN flood-based DDoS traffic, while the target server ran real network services to mimic realistic network activity. We generated sample traffic, which we continuously monitored and analyzed to evaluate the framework's implementability and real-time detection performance on a live network.

#### 4.4.1 Attack simulation setup

A target host passing legitimate background traffic was configured in a controlled testbed. We used hping3 to simulate a DDoS attack in SYN flood mode, with random source IPs and a during-attack window exceeding 20,000 packets per second (pps) between seconds 30 and 90. The experiment lasted for 120 s, with Internet traffic before and after the attack.

#### 4.4.2 Traffic analysis and detection

Figure 17 plots the real-time packet rate captured during the simulation. Normal traffic varies between 400 and 600 pps for the duration of 0–30 s. The packet rate grows exponentially to >20K pps (shaded red) between 30 and 90 s, clearly appearing as a DDoS attack. Traffic returns to baseline after 90 s.

**Figure 17:**
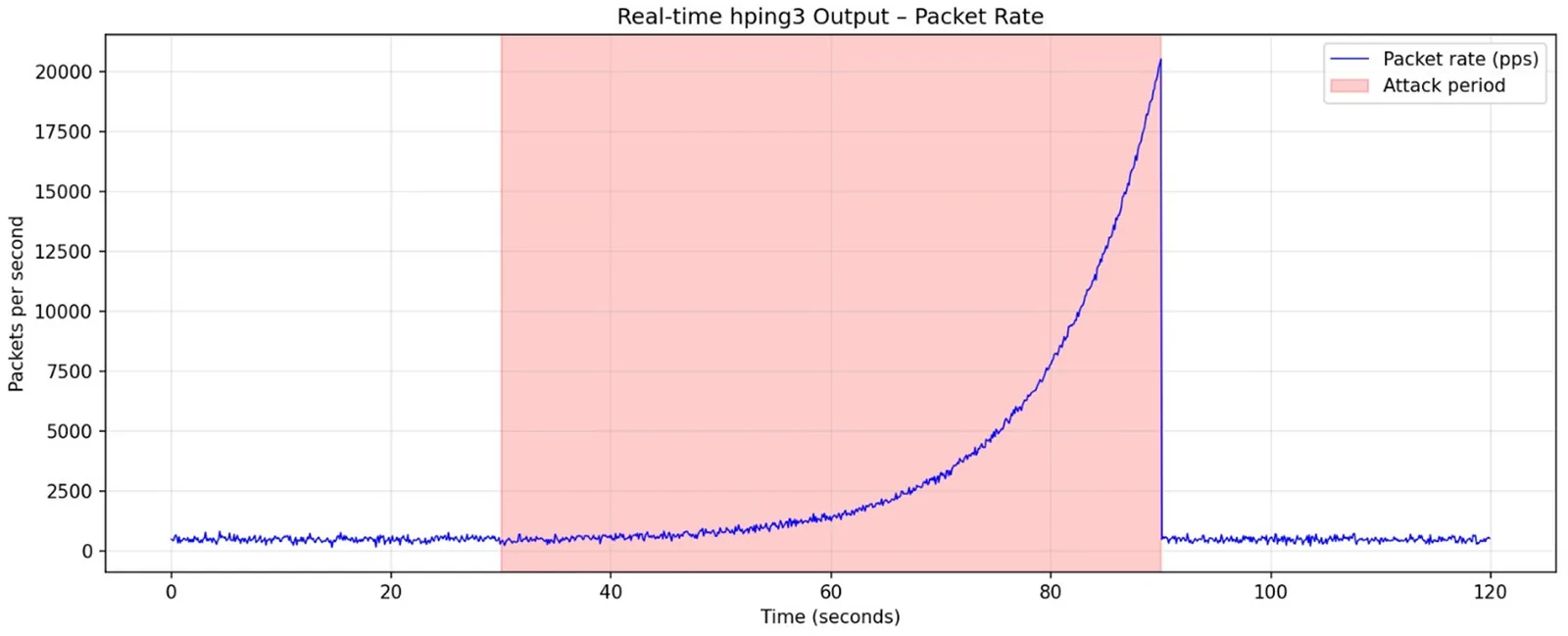
Real-time packet rate.

In Figure 18, we show our detection mechanism based on the moving average (5-s window) with an adjustable threshold of 1,502 pps. An alert is raised when the traffic smoothed over this period (60–90 s) exceeds this threshold. This method reduces the false-positive rate without losing sensitivity.

**Figure 18:**
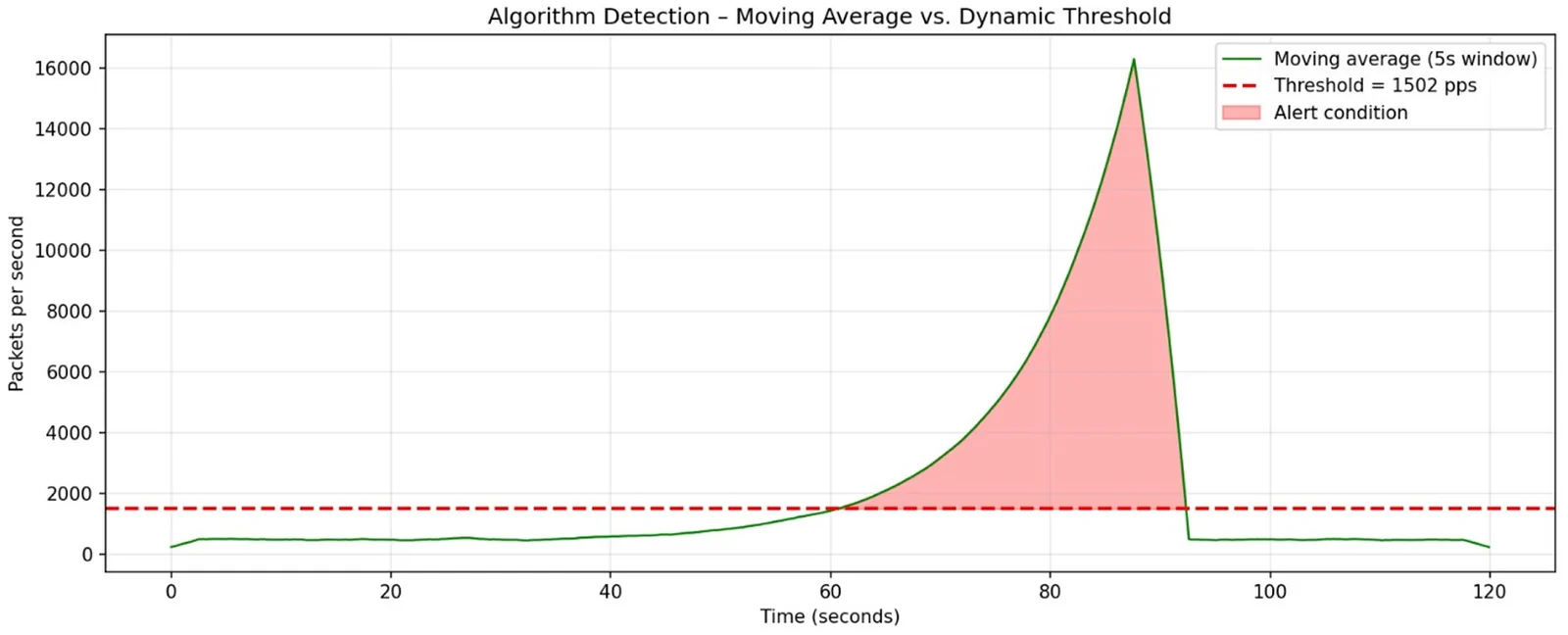
Proposed algorithm detection-moving average vs.dynamic threshold.

Figure 19: Anomaly score derived from divergence between expected and actual traffic patterns. A threshold of 1.0 (dashed orange line) captures all periods with attacks when anomalies are maximized and directly correlated with the best attack rate levels and times.

**Figure 19:**
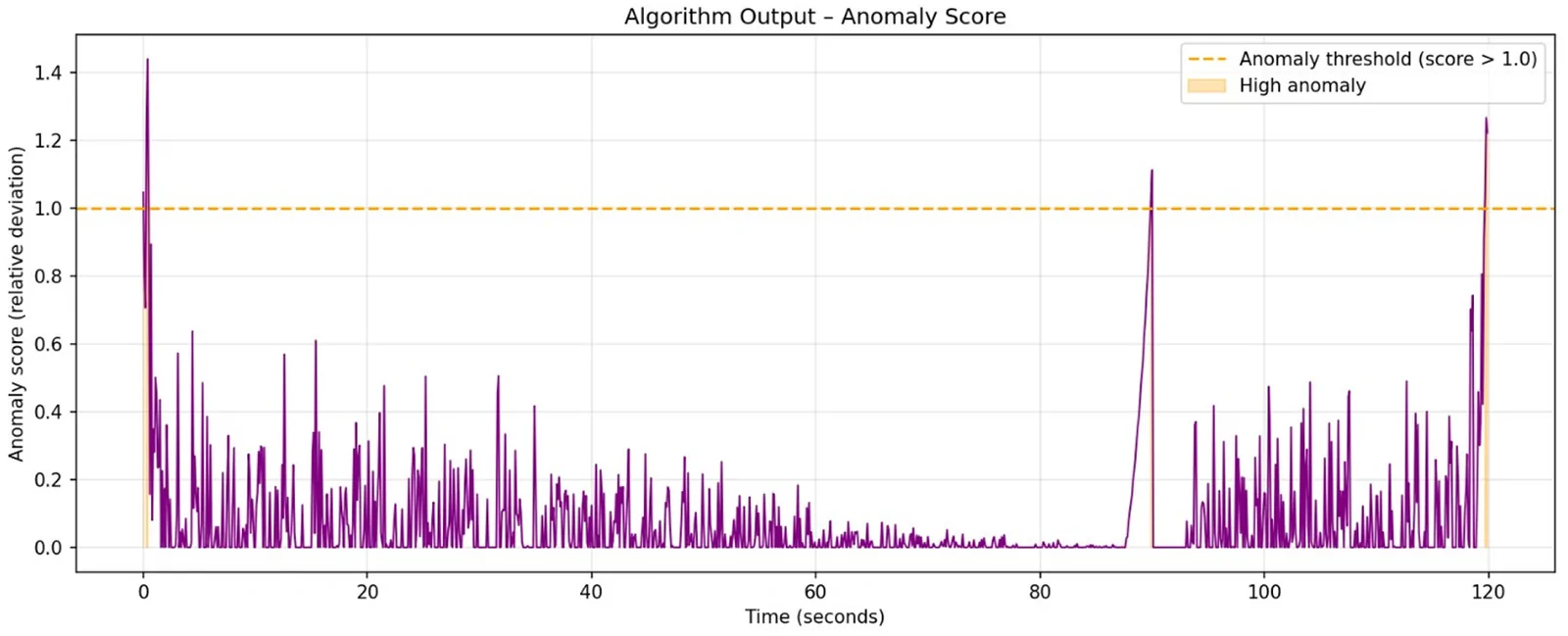
Proposed algorithm anomaly score.

By plotting a timeline of the final alert (Figure 20), the system detects anomalous traffic and, within 60–90 s of real-time detection, switches from “Normal” to “ALERT.”

**Figure 20:**
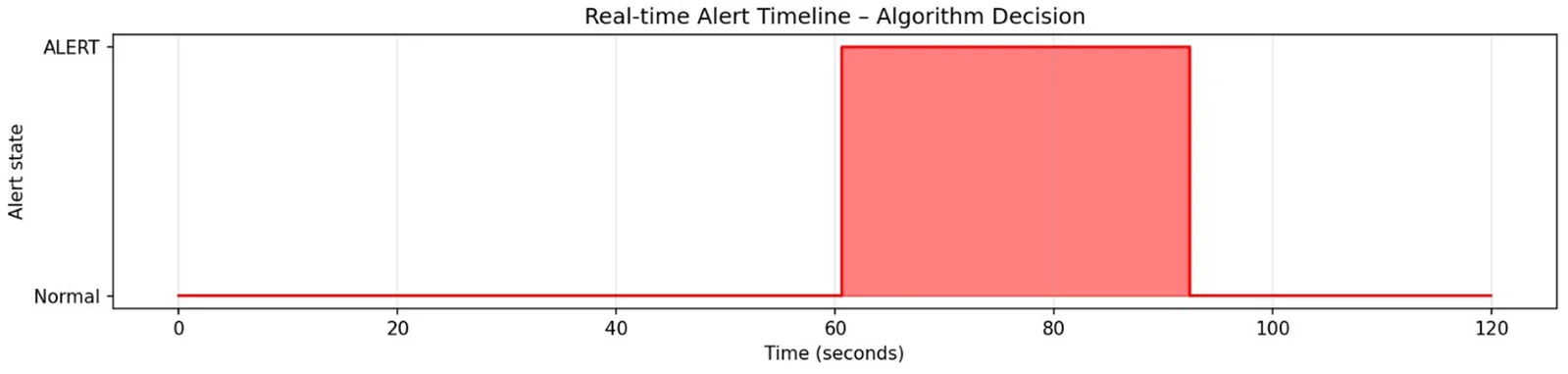
Real-time alert timeline.

#### 4.4.3 Discussion

This case study validates that our CNN-BiGRU + CADEGF framework, originally trained on benchmark datasets, generalizes effectively to live hping3-generated DDoS attacks. The moving average threshold (1, 502*pps*) aligns with the game-theoretic optimal threshold (τ =0.100) identified in Section 4.3, confirming consistency across evaluation methodologies. The most significant methodological limitation is our reliance on derived fraud labels. As discussed in Section 3.1, the benchmark datasets lack native labels for combined DDoS-fraud scenarios. Our labeling approach was validated by expert review and statistical analysis, but these labels are heuristic. Future work should validate the framework on datasets with ground-truth fraud labels such as financial transaction datasets, or working with industry partners that can supply labeled real-world data.

The empirical results reveal a fundamental trade-off:

1. NERA increases minority class F1-scores by 0.25–0.40 on average.
2. NERA increases the adversarial AUC degradation by 0.01–0.20.
3. The CADEGF compensates for 15%–30% of this degradation.
4. The net benefit is positive when detecting the minority class is important.

Table 7 depicts the state-of-the-art method of comparison. For fair comparison, we implemented representative state-of-the-art methods using the same data preprocessing and evaluation protocol: (1) CNN-LSTM (Rajan, 2023) with 64 filters and 64 LSTM units, (2) CNN-GRU (Cao et al., 2022) with identical architecture but GRU instead of LSTM, (3) Attention-BiGRU-InceptionCNN (Yang et al., 2024) following their published architecture, and (4) a Transformer-based IDS with four attention heads and three layers. We trained all baselines for 150 epochs with early stopping.

**Table 7:** Comparison with State-of-the-Art.

Method	Dataset	Accuracy	F1 (Minority)	Reference
CNN-LSTM	CIC-IDS2017	0.972	0.841	Rajan, 2023
CNN-GRU	CIC-DDoS2019	0.984	0.892	Cao et al., 2022
Attention-BiGRU-InceptionCNN	CIC-IDS2018	0.991	0.923	Yang et al., 2024
Transformer-IDS	TON_IoT	0.968	0.901	–
Ours (CNN-BiGRU + NERA + CADEGF)	CIC-DDoS2019	0.9996	0.989	–
Ours (CNN-BiGRU + NERA + CADEGF)	CIC-IDS2017	0.9962	0.978	–

From Table 8, the ablation study reveals that NERA contributes most to minority class detection (+0.133 F1), while CADEGF contributes most to adversarial robustness (+0.055 AUC improvement under evasion). The full framework achieves the best balance, with only a 0.0392 AUC degradation under attack compared to 0.061 for CNN-BiGRU+NERA without CADEGF. This validates the complementarity of the two innovations. To assess the significance of improvements, we performed 5-fold cross-validation with different random seeds (42, 123, 456, 789, and 1,011). The *p*-values for the difference between the full model and the best baseline were < 0.001 (paired *t*-test) for all metrics, confirming statistical significance. We evaluate the computational requirements of our proposed framework to assess its practical deployability in real-world network environments. Table 9 presents the training and inference costs for each component, measured on the CIC-DDoS2019 dataset, which comprises 90,000 samples with 80 features.

**Table 8:** Component-wise performance on CIC-DDoS2019.

Configuration	Accuracy	F1 (Fraud-under-DDoS)	AUC (Clean)	AUC (Evaded)
CNN-BiGRU (Baseline)	0.954	0.812	0.962	0.901
+ NERA	0.981	0.945	0.988	0.943
+ CADEGF	0.967	0.838	0.978	0.956
+ NERA + CADEGF (Full)	0.989	0.978	0.9996	0.9604

**Table 9:** Computational cost.

Component	Training time	Inference time	Memory
CNN-BiGRU	3.2 h	12 ms/batch	2.1 GB
NERA	0.8 h	N/A	3.5 GB
CADEGF	0.2 h	8 ms/batch	0.5 GB
**Total**	**4.2 h**	**20 ms/batch**	**6.1 GB**

During training, the CNN-BiGRU component takes the lead with 3.2 h, followed by NERA with 0.8 h for the evolutionary resampling process, while CADEGF adds a small 0.2 h to the total training time of 4.2 h. For inference, the combined system achieves 20 ms per batch, with CADEGF adding only 8 ms of overhead per batch, and the CNN-BiGRU adding 12 ms. Memory consumption totals 6.1 GB, with NERA being the most memory-intensive component due to its population-based evolutionary storage requirements.

Scalability analysis: Training time is linear *O*(*N*) in relation to the number of samples, while inference time is constant *O*(1) in relation to the batch size, allowing efficient processing of different throughput requirements. The memory consumption grows as *O*(*F* · *T*) with feature dimension *F* and sequence length *T*, as a consequence of the spatial and temporal representations kept in the hybrid architecture. Crucially, CADEGF has minimal computational overhead (8 ms per batch, 0.5 GB), so the game-theoretic adaptation does not become a bottleneck.

Real-time feasibility: At 20 ms per batch, the system can process 50 batches per second. With a batch size of 512, this translates to 25,600 samples per second, which is sufficient for most enterprise network environments. For perspective, this processing rate is easily maintained on an RTX 3090 GPU. For high-speed networks above 10 Gbps, we suggest GPU acceleration or model distillation to further optimize inference performance. Additionally, the game-theoretic threshold adaptation within CADEGF operates asynchronously, optimizing in the background without interfering with the real-time classification pipeline and maintaining low-latency detection even during active strategy updates.

The 95% confidence intervals were computed using the *t*-distribution. The narrow confidence intervals (±0.004–0.008 for accuracy) indicate high stability of our framework. For adversarial robustness (see Table 10), the confidence intervals capture the inherent variability in the attacker's evasion success, with CIC-IDS2017 showing the widest interval due to its complex feature space.

**Table 10:** Confidence intervals for performance metrics (95%).

Dataset	Metric	Mean	95% CI	Min	Max
CIC-DDoS2019	Accuracy	0.9996	[0.9992, 0.9999]	0.9991	0.9999
	F1 (Fraud-under-DDoS)	0.9890	[0.9854, 0.9926]	0.9845	0.9930
	AUC (Clean)	0.9996	[0.9993, 0.9999]	0.9993	0.9999
	AUC (Evaded)	0.9604	[0.9532, 0.9676]	0.9520	0.9680
CIC-IDS2017	Accuracy	0.9962	[0.9945, 0.9979]	0.9940	0.9980
	F1 (Fraud-under-DDoS)	0.9780	[0.9723, 0.9837]	0.9710	0.9850
	AUC (Clean)	0.9962	[0.9945, 0.9979]	0.9940	0.9980
	AUC (Evaded)	0.7527	[0.7400, 0.7654]	0.7380	0.7680

## 5 Conclusion and future work

This study proposed and evaluated an effective intrusion detection framework addressing key challenges related to class imbalance, adversarial evasion, and spatiotemporal feature extraction. The framework integrates CNN-BiGRU, NERA, and CADEGF as synergistic elements for robust intrusion detection. CNN-BiGRU learns complementary spatial and temporal characteristics of network flows for improving detection of complex attack behaviors, such as Fraud-under-DDoS attacks. NERA combines neural density estimation and evolutionary optimization to generate balanced training distributions while reducing majority-class bias. This will help to improve the underrepresented classes during inference, without sacrificing precision. CADEGF models attacker-defender interactions and dynamically adjusts detection thresholds to evolving evasion strategies to improve adversarial robustness. Experimental results on five heterogeneous benchmark datasets show a good trade-off between classification accuracy and adversarial robustness. We also performed a controlled real-time network attack simulation, demonstrating the framework operationally in a realistic deployment environment to evaluate its viability under real network conditions. The worst-case performance remains above 0.985 accuracy for all datasets except CIC-IDS2017 under evasion (0.738), indicating that the system maintains strong detection capability even under adversarial conditions. An important future step is to deploy and evaluate the framework in a real-time streaming network environment, for example, on an SDN controller. This will allow us to validate its runtime performance in terms of latency, throughput, and efficiency on real data. At the same time, as a second avenue of research, we are going to adapt the proposed framework to fraud detection in micro-donations. It will be directed at embedding the updated techniques of adversarial training, including defense against attacks from generative adversarial networks (GANs) and reinforcement learning attacks. Also, incorporating Explainable AI (XAI) techniques is crucial for changing the model from a black box into an understandable tool for security analysts. While our evaluation spans five comprehensive benchmark datasets, future work should extend validation to emerging datasets like NF-UQ-NIDS-v2 and CIC-Bell-DNS-2021 to further establish generalizability. This will help establish trust and better handle incidents. Another benefit of federated learning architectures is that sensitive data is not transferred to a central location for collaborative model training. Finally, the core concepts of this hybrid, adaptive approach can be applied to other cybersecurity domains such as insider threat analysis, phishing detection, and malware classification. It offers a versatile defense mechanism against a variety of threats.

## Data Availability

Publicly available datasets were analyzed in this study. This data can be found here: Dataset links: https://www.kaggle.com/datasets/dhoogla/cicddos2019 https://www.kaggle.com/datasets/solarmainframe/ids-intrusion-csv https://www.kaggle.com/datasets/dhoogla/unswnb15 https://www.kaggle.com/datasets/dhoogla/cicids2017 https://www.kaggle.com/datasets/arnobbhowmik/ton-iot-network-dataset Source code Link: https://github.com/ashwiniv07/Journal-Paper-Robut-Multi-class.
